# Unveiling the biochemical potential of *Acacia jacquemontii* as a therapeutic agent in parkinson’s disease: A multi-model *in Vitro, In Vivo, and In Silico* Study

**DOI:** 10.1371/journal.pone.0334312

**Published:** 2026-02-19

**Authors:** Andleeb Asghar, Tahir Ali Chohan, Aisha Qayyum, Sibghat Mansoor Rana, Khuram Ashfaq, Hafiz Muhammad Mazhar Asjad, Talha Ali Chohan, Abdulwahab Alamri, Ahmed Alsolami, Fawwaz F. Alshammrie, Hammad Saleem, Sirajudheen Anwar

**Affiliations:** 1 Institute of Pharmaceutical Sciences (IPS), University of Veterinary and Animal Sciences (UVAS), Lahore, Pakistan; 2 Department of developmental and behavioral pediatrics, University of Child health sciences, Lahore, Pakistan; 3 University college of pharmacy, University of the Punjab, Lahore, Pakistan; 4 Faculty of pharmaceutical sciences, Lahore university of biological and applied science,; 5 Department of Pharmacy, Forman Christian College University Lahore, Lahore, Pakistan; 6 Institute of Molecular Biology and Biotechnology, The University of Lahore, Lahore, Pakistan; 7 Department of Pharmacology and Toxicology, College of Pharmacy, University of Ha’il, Ha’il, Saudi Arabia; 8 Department of Internal Medicine, College of Medicine, University of Ha’il, Ha’il, Saudi Arabia; 9 Department of Dermatology, College of Medicine, University of Ha’il, Ha’il, Saudi Arabia,; 10 Saveetha Dental College and Hospitals, Saveetha Institute of Medical and Technical Sciences (SIMATS), Saveetha University, Chennai, Tamil Nadu, India; Kwara State University, NIGERIA

## Abstract

Parkinson’s disease (PD) is a progressive neurodegenerative disorder marked by oxidative stress, inflammation, and the degeneration of dopaminergic neurons. Current treatments focus more on symptom management rather than disease prevention. *Acacia jacquemontii*, rich in antioxidants, may offer a novel therapeutic approach for PD. This study aims to investigate the phytochemical composition, antioxidant capacity, anti-Parkinsonian efficacy, and *in-silico* validation of *Acacia jacquemontii* methanol extract (AJME) using liquid chromatography-mass spectrometry (LC-MS). Secondary metabolites were identified, and total alkaloid, phenolic, and flavonoid contents were quantified. LC-MS was used for detailed compound profiling. Antioxidant activity was evaluated using the DPPH assay. *In vivo* tests on Wistar rats modeled PD through haloperidol administration. AJME’s anti-Parkinsonian effects were assessed via histological, biochemical, and behavioral analyses. *In-silico* techniques, including molecular docking, structural interaction fingerprinting, ADME prediction, DFT, MESP studies, and molecular dynamics (MD) simulations, were employed to understand AJME molecules’ binding interactions and electronic properties. *In vivo*, AJME improved locomotor activity, memory, exploratory behavior, oxidative stress markers (SOD, CAT, GSH, MDA), and neurotransmitter levels (dopamine, noradrenaline, serotonin) in rats. *In-silico* validation identified CP21 as a potent ligand. MD simulations indicated stable AJME-AChE complexes, with enhanced binding affinity through hydrophobic and van der Waals interactions. *A. jacquemontii* exhibits significant phytochemical, antioxidant, and anti-Parkinsonian properties. The combined *in vitro, in vivo*, and *in silico* studies, supported by LC-MS analysis, suggest that AJME could provide a promising option for developing new therapeutic approaches for PD. However, clinical evaluation is necessary to establish its efficacy and safety in human subjects.

## 1. Introduction

Neurological disorders are related to the nervous system, which is comprised of the brain, the nerves, and the spinal cord [1]. Motor neuron degeneration and Parkinson’s disease are the outcome of a multifaceted process that includes numerous elements and phases. These include the regulation of protein homeostasis, neuronal dysfunction, inflammation of the neurological system, apoptosis, and oxidative stress. When these factors work in combination, they lead to the progressive loss and degeneration of nerve cells [2]. According to WHO (World Health Organization) data, Parkinson’s disease is common in Pakistan, with an estimated 450,000 patients. It is a severe health hazard, with a prevalence of approximately 1% in the population [3]. PD is a progressive neurodegenerative disorder characterized by the degeneration of dopaminergic neurons in the substantia nigra pars compacta and the nigrostriatal pathway. This disorder, originally described by Dr. James Parkinson in 1817 in his work “Essay on the shaking palsy,” exists in two primary forms: familial that is genetic and sporadic which is idiopathic [4]. It is a chronic disease that evolves gradually and is characterized by both motor and non-motor symptoms that include resting tremor, postural and gait instability, rigidity, and slowness of movement (bradykinesia) [5]. Parkinson’s disease has some of the characteristics common with other neurodegenerative diseases, such as the presence of Lewy bodies, which are accumulations of α-synuclein-rich proteins associated with protein degradation [6]. These abnormal bodies together with a decline in dopaminergic neurons in the striatum leads to the impairment of voluntary motor functions, then spread throughout the brain to the neocortical and cortical areas as Parkinson’s disease progresses [7].

Typically, the onset of PD tends to manifest in individuals aged between 65 and 70, with a higher incidence observed among men compared to women [8]. The development of PD is a complex interplay of various factors, encompassing both genetic and non-genetic elements, along with environmental influences [9]. Key pathological processes include the accumulation of protein deposits, damage to mitochondria, impaired pathways for clearing proteins, inflammation within the nervous system, oxidative stress, excitotoxicity, and genetic mutations [10]. Genetic factors contribute to a mere 5–10% of PD cases, where specific gene variations impact diverse molecular pathways, ultimately contributing to the dysfunction observed in Parkinson’s disease. Comprehensive studies examining the entire genome, known as genome-wide association studies (GWASs), have pinpointed specific proteins encoded by genes connected to these pathways, especially in cases of sporadic PD [11]. These findings are particularly pertinent to abnormalities in mitochondrial function and inflammation within neuronal cells. Current approaches to treating PD primarily focus on mitigating its symptoms, lacking the capacity to impede the disease’s advancement or safeguard the decline of dopaminergic neurons [12].

In light of these limitations, therapeutic approaches are tailored based on the patient’s age and specific symptoms. For younger patients, dopamine agonists are commonly prescribed, while older patients tend to benefit more from levodopa [13]. In cases where initial motor fluctuations occur, MAO-B inhibitors are recommended as an initial therapy, and for those experiencing wearing-off symptoms, COMT inhibitors can be employed to enhance the effectiveness of levodopa [14]. However, pharmacological treatments do not remain the only cure for such diseases; deep brain stimulation has been proved to be one of the most effective treatments [15,16]. The management of PD has placed a greater emphasis on improving the overall quality of life for patients. Future strategies are likely to revolve around two key aspects [17]. Firstly, there will be a focus on identifying individuals who are at a high risk of developing PD, allowing for early intervention and potentially slowing down the disease’s progression. Secondly, efforts will be directed towards developing innovative formulations of existing medications. An example of this is *β*-asarone, a compound that has displayed promise in the treatment of PD, with the potential to enhance the efficacy of current medications while minimizing their side effects [18].

Numerous types of plants have been recognized for their promising potential in addressing neurodegenerative conditions. These plants provide a range of protective properties that help counteract the process of neurodegeneration [19]. Plant species with antioxidant properties are particularly recognized for their ability to ameliorate the disease process. *A. jacquemontii*, which contains potent antioxidants like quercetin, has been acknowledged for safeguarding neurons against cellular degeneration associated with free radicals in PD [20]. Additionally, numerous bioactive compounds isolated from therapeutic plants have demonstrated efficacy in alleviating neurodegenerative disorders.

*A. jacquemontii* belongs to the Fabaceae family and is also known as Bhu-banwali, Raati-banwali, Baonli and Bhunwali. It is a xerophytic shrub or tree, which can reach a height of about 2.5m [21]. This plant boasts rigid and smooth zigzag branches, conspicuous peduncular joints, sharp-pointed stipules, and hairless ovaries. Its small, wide pods measure around 5–7.5 cm in length and 8–17 mm in width, containing straight, compressed seeds held together by sutures (typically 5–6 seeds per pod) [22]. *A. jacquemontii* exhibits rapid growth, is easily coppiced, and enriches arid ecosystems by contributing nitrogen. Its taproot enables it to access water from deeper soil, ensuring it remains green even during dry seasons [23]. Studies have shown that the leaves of this species contain approximately 22% crude protein, while the seeds contain approximately 33% crude protein, with varying crude fiber contents of 49% and 15% for leaves and seeds, respectively [24].

Parkinson’s disease remains a global health concern as it is marked by the degeneration of dopaminergic neurons and indications of oxidative stress and inflammation in the nervous system. There is currently no cure or therapy that can halt the progression of the disease. *A. jacquemontii* was selected for this study because it contains polyphenols, flavonoids and alkaloids which are known to be anti-inflammatory and to help with antioxidants. They have shown protection for the nervous system, so *A. jacquemontii* seems useful for treating the disease, as the current medicines only manage the symptoms of Parkinson’s disease. This study aims to identify the phytochemicals, antioxidant potential, and antiparkinsonian properties of *A. jacquemontii*, with an *in-silico* validation in response to the demand for better therapeutic remedies for PD.

## 2. Materials and methods

### 2.1. Plant collection and extraction

The Cholistan Institute of Desert Studies, The Islamia University of Bahawalpur, confirmed the plant’s authenticity after the leaves of *A. jacquemontii* were purchased at the local market in Bahawalpur, Pakistan. The voucher number used for authentication was CIDS/IUB-1901/63. The leaves were then washed, left to dry under shade and ground into a coarse powder using an electrical mill. A total of 2 kilograms of shade-dried *A. jacquemontii* leaves were ground into coarse powder before being macerated in 5 liters of 80% methanol solution. This maceration lasted 7 days at a temperature of 25°C. Methanol was chosen as the solvent because of its established efficiency in extracting polyphenols and flavonoid secondary metabolites from plant material. After the maceration procedure, the resultant solvent phase was separated, and any leftover solid residue was treated to a second round of maceration to guarantee thorough extraction. After undergoing two rounds of maceration, the combined filtrates were efficiently concentrated utilizing a Heidolph USA rotary evaporator at reduced pressure conditions. Following this concentration step and subsequent drying, we achieved a 50-gram yield of a viscous solid substance as the methanol extract.

### 2.2. Phytochemical composition

#### 2.2.1. Preliminary qualitative phytochemical analysis.

Preliminary screening identified many secondary metabolites in the extract. The details of these tests are summarized in supplementary material **(**S3 Table***).***

#### 2.2.2. Estimation of total bioactive contents.

The total bioactive contents including Total Alkaloids contents, Total Phenolic Content (TPC), and total flavonoid content (TFC) was determined using well-established previous methods. The total alkaloids in the extract were expressed as milligrams of atropine equivalents (AEqs/g). The results of TPC were expressed in milligrams of GAE/g, and the values were expressed in terms of mg QE/g of DW.

Two milliliters of concentrated H_2_SO_4_, one milliliter of gallic acid in 5% methanol, and a sample or conventional alcoholic solution were all contained in a test tube. After ten minutes of boiling the solution, the absorbance at 660 nm was determined. The test tube holding the standard contained all of the necessary reagents; the sample was the only thing missing. Atropine was used to produce the standard curve, and standard solutions containing 10, 20, 30, 40, 50, and 60 μg/mL were prepared. The total alkaloids in the extract were expressed as milligrams of atropine equivalents (AEqs/g).

Total Phenolic Content (TPC) was determined by using the Folin-Ciocalteu reagent. Consequently, the absorbance of the reaction mixture was recorded at 760 nm and the results were expressed in milligrams of GAE/g. The aluminum chloride colorimetric method was also employed in order to estimate the total flavonoid content (TFC). At 510 nm in wavelength, the absorbance was determined, and the values were expressed in terms of mg QE/g of DW.

#### 2.2.3. Liquid chromatography mass spectrometer (LC-MS) analysis.

For the LC-MS analysis, an UltiMate 3000 RS liquid chromatography system equipped with a MicrOTOF-Q II mass spectrometer was used as reported previously. For the LC-MS analysis, an UltiMate 3000 RS liquid chromatography system equipped with a MicrOTOF-Q II mass spectrometer was used. Separation was achieved using a C18 Ascentis Express column with a mobile phase of acetonitrile and 0.1% formic acid at a flow rate of 0.3 mL/min with a preset gradient. The autosampler and column had temperature settings of 15°C and 20°C, respectively. Standard samples were injected twice, while test samples were injected three times, each at a volume of three μL. The ESI + ion source operated at 180°C and 6 kV of voltage. The nebulizer gas pressure was 0.3 bar, and the dry gas flow rate was 7 L/min. The mass spectrometry study was performed in full scan mode (m/z 150–1000) with different impact energies. Compounds were identified using Untargeted Auto-MS/MS mode with a 20-eV collision energy, and the findings were confirmed using Multiple Reaction Monitoring (MRM) mode. The TOF detector was calibrated using isopropanol-sodium formate clusters before to each operation [25]. Chromatographic separation was performed using a C18 column with a mobile phase of acetonitrile and water (both containing 0.1% formic acid) in gradient mode. The mass spectrometer was operated in positive ion mode, with a scan range of 50–1000 m/z for compound identification [25].

### 2.3. *In-vitro* biological assays

#### 2.3.1. Determination of antioxidant activity using DPPH assay.

The reduction of the DPPH radical cation by the *A. jacquemontii* methanol extract (AJME) was assessed through a DPPH assay. In this assay, a 96-Well microplate was used and in each well, 100μl of the solution was prepared which consisted of 10μl of the test solution and 90μl of the DPPH solution. The mixture was then shaken and allowed to stand at 37°C for 30 minutes to allow the reaction to occur. The decrease of absorbance at 517 nm, which represents the DPPH radical scavenging activity, was measured using a Synergy HT microplate reader from Biotech, USA. In the study, ascorbic acid was employed as the standard antioxidant [26]. Each of the experiments was done three times to reduce the chances of errors and to increase the level of reproducibility. The IC50 values, which is the concentration that inhibits 50% of DPPH radicals, were determined using dilution factor with the help of Ez-fit5 software from Amherst USA and Perrella Scientific Inc. The percentage inhibition of the DPPH radical was calculated using the following formula:


$$Inhibition\;(\%)=\frac{(Absorbance\;of\;control-absorbance\;of\;test\;solution)\;\times \text{1}\text{0}\text{0}}{Absorbance\;of\;control}$$ (1)

Where

Absorbance of Control = Total radical activity without inhibitor

Absorbance of Test = Activity in the presence of test compound

#### 2.3.2. In vitro acetylcholinesterase assay.

The effect of AJME on AChE inhibition was determined using a modified method from the previous studies. The total volume of the reaction mixture was 100 μL, and the ingredients included 10 μL of the test drug (0. 7 50 mM Na2HPO4 buffer and 10 μL of AChE enzyme (0. 005 unit/well). The absorbance of the mixture at 405 nm was taken pre reading before the mixture was well mixed. The reaction was started by adding 10 μL of DTNB (0. 5 mM/well) and 10 μL of the substrate, acetylthiocholine iodide (0. 5 mM/well) to each well after 10 minutes of incubation at 37°C. The absorbance of the reaction mixture was then measured again at 405 nm after 30 minutes of incubation at 37°C using the Synergy HT microplate reader (Biotek, USA). All the experiments were performed in triplicates and eserine at a concentration of 0. 5 mM/well was used as the positive control. The percentage of AChE activity inhibition was determined by the following formula:


$$\;Inhibition\;(\%)=Control-\frac{Test}{Control}\times \text{1}\text{0}\text{0}$$ (2)

where “Test” represents the enzyme activity in the presence of the test substance and “Control” represents the total enzyme activity in the absence of the inhibitor. Perrella Scientific Inc., Amherst, USA developed EZ-Fit Enzyme Kinetics software which was used to determine the IC50 values, which is the concentration at which 50% of the enzyme activity is inhibited.

### 2.4. *In-vivo* anti-parkinson activity

#### 2.4.1. Experimental animals.

A group of rats with weights ranging from 150 to 200 grams, regardless of gender, were used to test *in vivo* anti-Parkinson activity. These rats were purchased from the Animal House of the University of Veterinary and Animal Sciences, Lahore, Pakistan and kept under reasonable conditions, including polypropylene cages in an animal housing with light-dark cycle of 12h and a room temperature of 25°C. After 7 days of feeding and providing the animals with ad libitum access to water, the animals were randomly divided into six groups. All animal experiments were approved by the Institutional Review Board of the University of Veterinary and Animal Sciences, Lahore (Ethical approval no. DR/773), in accordance with guidelines from the National Research Council (1996). This clearance was provided in accordance with the regulation of the National Research Council’s Institute of Laboratory Animal Resources, which was commissioned in the field of life sciences in 1996.

#### 2.4.2. Disease induction.

Parkinson’s disease was developed in the rats, except the normal control group, and haloperidol was given at a dose of 1 mg/kg via intraperitoneal injection once daily for 21 consecutive days. In addition, one hour prior to the start of extract therapy, haloperidol was administered through an injection. The use of the haloperidol-induced Parkinson’s model in this study is appropriate for simulating PD-like symptoms, particularly motor dysfunction and catalepsy. However, while models like 6-OHDA or MPTP are more pathophysiologically relevant, the haloperidol model was chosen due to its well-established ability to induce consistent motor deficits, making it a reliable tool for screening potential therapeutic agents [27].

#### 2.4.3. Study design.

In this investigation thirty-six healthy albino Wistar rats of both sexes aged around seven weeks were used. Both sexes were equally represented. The rats were divided into six groups of six rats each (6 rats/group). Experimental groups and the respective treatments used in the Parkinsonian model study are described in supplementary table (S4 Table**).**

The three doses of AJME (200, 400 and 600 mg/kg) were chosen from preliminary data and based on the guidance of previous studies where similar concentrations were utilized for extracts from different plants [27–30]. The doses used were within the range of potential therapeutic effects, from the lower to the higher concentrations. Although limited research has been conducted specifically on *A. jacquemontii* in the context of Parkinson’s disease, studies on plants from the same *Acacia* family have employed similar dosing regimens. This suggests that the doses used in the present investigation are in line with those typically tested for neuroprotective effects in Parkinson’s models. This provides a solid basis for extending the research on *A. jacquemontii* and exploring its therapeutic potential, particularly with regard to its antioxidant and neuroprotective properties. The consistency in dosing across studies involving related plants strengthens the rationale for the chosen dosages and paves the way for further exploration of *A. jacquemontii* in treating Parkinson’s disease.

Each group completed a sequential 21-day cycle of therapy. Behavioral and weight changes were noted at the start, mid, and end of the research phase. For biochemical and histological study, all the rats were sacrificed at the end of the 21-day treatment period. To assess the oxidative stress and quantify the concentrations of dopamine, serotonin, and norepinephrine, the rats were sacrificed by decapitation, and their brains were rinsed in normal saline and immediately immersed in ice-cold phosphate buffer (pH 7.4). Blood samples were drawn through cardiac puncture. Before histopathological examination, the brains were fixed in 10% buffered formalin.

#### 2.4.5. Behavioral analysis.

The Open-Field Test, Catalepsy Test, Hole Board Test, Narrow Beam Walk Test, Y-Maze Test, and Swim Test were used to conduct behavioral analysis.

In the behavioral analysis, parameters such as the number of squares crossed and central explorations (locomotor and exploratory behavior) were recorded in the open-field test; latency (time to initiate movement) and cataleptic reaction duration were measured in the catalepsy test on Days 7, 14, and 21 post-treatment; hole probing (curiosity behavior) and climbing/rearing activities (exploratory behavior) were noted in the hole board test; latency and foot errors were assessed in the narrow beam walk test on Day 21; spontaneous alternation behavior (short-term memory) was evaluated in the Y-maze test on Day 21; and swimming scores and immobility time were measured in the swim test on Days 20 and 21 post-treatment.

2.4.5.1. Open-field test: The locomotor and exploratory activity of the experimental rats was assessed using an activity meter developed for this purpose. This apparatus was made from a wooden box that was square in shape and measured 100 cm on each side of the box and 45 cm in height. The inside of the box was painted white and the ground of the box was further divided into twenty-five squares by black lines. The arena was sanitized with ethanol between trials to prevent olfactory bias. Each rat was put into the center of the box and the box was left open for the rat to move around for five minutes. During this period, several parameters were recorded, including the number of central and peripheral square crossings, duration of time spent in different areas, instances of defecation, freezing behavior, and various postures. “Freezing behavior” refers to a temporary inability to move, often seen in Parkinson’s disease as a result of motor dysfunction. It is associated with a disruption in the brain’s movement control. “Various postures” involve abnormal body positioning, such as rigidity or stiffness, which also indicates impaired motor coordination and muscle control, common in neurodegenerative disorders like Parkinson’s disease.

2.4.5.2. Catalepsy assessment: To induce catalepsy in rats, haloperidol was administered, resulting in unresponsiveness to external stimuli and muscular stiffness. Following administration, each rat was placed with its forelimbs on a raised wooden bar, which had a diameter of 1 cm and was set at varying heights between 3–9 cm. The time it took for the rats to spontaneously correct this forced posture was recorded as an indicator of catalepsy. The cataleptic state was considered resolved when the rats either climbed off the bar or made contact with the floor.

Observations were systematically documented at intervals of 30, 60, 90, and 120 minutes within a controlled environment maintained at 23-25^o^C, with a maximum observation period of 5 minutes. The catalepsy rating system included three specific criteria:

1. A score of 0 was assigned if the rats moved normally when placed on the table.
2. A score of 0.5 was given if the rats responded appropriately to being pushed or touched.

A score of 2 was issued if the rats did not adjust their posture within 10 seconds, with an additional point added for each paw that failed to correct its position.

This scoring system provided a detailed measure of the severity of catalepsy induced by haloperidol.

2.4.5.3. Hole board test: The hole board test was used to assess the behavioral components of the experimental animals. The gadget measured 30 centimeters by 30 centimeters and had 16 holes 16 centimeters apart. On the twentieth day of treatment, each animal was given thirty minutes to acclimate to the area around the apparatus before being placed in the center for evaluation. A 120-second experiment revealed a variety of exploratory behaviors, such as edge sniffing, head dipping, strolling, immobile sniffing, climbing, rearing, and immobility for brief periods of time.

2.4.5.4. Narrow beam walk test: Rats were utilized in this study as a model of Parkinson’s disease to evaluate motor coordination and balance. The rats were made to navigate a 100-centimeter-long by 4-centimeter-wide hardwood plank for a duration of two minutes. Each rat’s balance and motor coordination were assessed by timing how long it took it to walk from one end of the board to the other.

2.4.5.5. Y-Maze test: The Y-maze test was employed in assessment of spontaneous alternation behavior in an attempt to quantify working short-term memory. The Y-maze consists of three equilateral arms (A, B, and C) each with a length of 35 cm and height of 25 cm and width of 10 cm. They are joined at a middle triangle. Rats were placed with their backs towards one of the two central arms. It was recorded as an entry when the rat used all the four paws to enter an arm. Alternation was defined as the successive visits to the three arms in a cycle (e. g., ABC, BCA or CAB) but not revisiting the arm that has been visited immediately before (e.g., CAC). This way, we were able to determine the maximum spontaneous alternation by taking into consideration the total arm entries. The percentage of spontaneous alternation was then obtained by multiplying the above result by 100 and the actual number of alternations by the maximum number of possible alternations. This test was aimed at ascertaining the extent to which the rats were able to exercise their short-term memory.

2.4.5.6. Swim test: The examination of depressive characteristics in animals, commonly known as the Behavior Despair Test, is carried out to assess the potential for immobility or mobility. An enclosure measuring 16 cm in height, 40 cm in length, and 25 cm in width is utilized, and it is filled with water. Throughout the test, a diligent focus is maintained on ensuring that the animal’s head remains above the water’s surface. The swimming test procedure was performed for two consecutive days, on the 20th and 21st day of the experiment. Each rat was then put in a separate cylindrical cage filled with water up to 19 cm deep and kept at 23°C temperature. During the test, each rat underwent a 6-minute swimming session, during which their swimming behavior was closely observed. The immobility time during the final four minutes of the session was recorded, preceded by a 2-minute acclimatization period.

#### 2.4.6. Evaluation of biochemical parameters.

The antioxidant enzymes like glutathione, superoxide dismutase, catalase, and the lipid peroxidation product malondialdehyde were measured in this study. Further, the levels of the enzyme acetylcholinesterase (AChE) and the concentration of dopamine, serotonin, and norepinephrine were determined in the homogenates of the brain. The assays for CAT, SOD, GSH, and MDA levels were done using standard methods that have been used in prior studies. These protocols ensured accurate and reliable measurement of the biochemical parameters in the brain samples.

#### 2.4.7. Measurement of nitrite levels.

The nitric oxide levels were determined by measuring the quantities of nitrite in the tissue samples using the Griess reagent test. By adding an equal proportion of Griess reagent to the tissue homogenate, a total volume of 36 ml of the solution was produced in this procedure. The mixture was incubated for ten minutes to ensure that the reaction happens. After incubation, the mixture’s absorbance at 546 nm was measured. The nitrite content was then determined by comparing the absorbance data with the regression line obtained from the nitrite calibration solutions.


                         absorbance (Y)=0.003432+ 0.0366 (3)

#### 2.4.8. Tests for the estimation of neurotransmitter levels.

To ascertain the quantities of neurotransmitters, a rather elaborate procedure was carried out, which began with the extraction of rat brains and weighing them after the sacrifice of the rats. The cerebral tissues were first subjected to homogenization, followed by treatment with 5 mL HCl-butanol solution and centrifugation at 2000 rpm for 10 minutes. A portion of the supernatant was taken and for 10 minutes, the sample was vigorously shaken with 0. 31 mL of 0. 1 M HCl and 2. 5 mL of heptane. Once more, this mixture was centrifuged for ten minutes at 2000 rpm with the temperature set at 0°C. The 0. 50 µL of the separated liquid phase was used to further determine the concentration of neurotransmitters. The liquid phase was heated to 100°C, 0. 2 mL of it was taken for the analysis. 25 mL of O-phthalaldehyde was added, and the mixture was then allowed to cool to room temperature. Serotonin level was quantified by fluorescence method at 305 nm excitation and 340 nm emission. To determine the concentrations of dopamine and noradrenaline, a 0. 50 µL of the aqueous phase was combined with 2 mL of the organic phase and then 2 mL aliquot of the aqueous phase was mixed with 0. 05 mL of 0. 4 M HCl and 0. 1 mL of 0. 1 M sodium acetate/ethylenediaminetetraacetic acid (EDTA) buffer, pH 6. 9. After that, 1. To start 5 minutes of oxidation, 0. 1 mL of Na2SO3 and 0. This is approximately 1 mL of acetic acid. The two compounds were mixed and heated at 100°C for six minutes, and then the mixture was cooled to the room temperature. The absorbance of noradrenaline and dopamine was determined at 450 and 350 nm, respectively.

#### 2.4.9. Acetylcholinesterase activity.

For this experiment, the phosphate buffer of pH 8 was prepared by diluting 6 mL of phosphate buffer with 100 μL of 0.1 M DTNB and 20 μL of acetylthiocholine iodide to create the reaction solution. Additionally, to the mixture 4 mL of tissue homogenate was added. The yellowish color of this mixture was the sign of the change that DTNB and acetylthiocholine iodide had brought. Subsequently, the absorbance at 412 nm was measured. Changes in absorbance were monitored at 2-minute intervals for a total duration of 10 minutes.

### 2.5. Histopathological analysis

Silver staining was employed to treat the brains of all animals and histopathological analysis was performed. According to the morphological changes, sections were scored as follows: 1) Neurofibrillary tangles score: 0 = No change, 1 = Sparse neurofibrillary tangles, 2 = Moderate neurofibrillary tangles, 3 = Severe neurofibrillary tangles; 2) Plague score: 0 = No change, 1 = Sparse plague, 2 = Moderate plague, 3 = Severe plague; 3) Combinatory sore: 1 + 1 = Sparse neurofibrillary tangles + Sparse plague.

### 2.6. *In-silico* studies

#### 2.6.1. Molecular docking.

Molecular docking, a common method for studying protein-ligand interactions, was employed here using the crystal structure of AChE and a compound called donapezil (PDB:4ey7) [31]. A computational method called molecular docking was used to forecast the ideal binding orientation and affinity between a biological macromolecule, like a protein or nucleic acid, and a tiny molecule, or ligand. This technique is essential to structural biology and drug discovery because it finds possible binding sites and evaluates whether ligand-macromolecule interactions are feasible. By simulating the intermolecular forces and energetics involved, molecular docking helps in understanding how stable complexes form between the ligand and its target, thereby providing insights into their interaction mechanisms. First, the protein structure underwent preparation steps that involved removing water molecules and filling in missing parts using a tool called Prime. Active site water molecules distant from the ligand were removed, followed by optimizing the structure. Ligand molecules were generated using LigPrep, producing various structures with different properties. These ligands were then optimized using the OPLS4 force field [32].

To define the docking search space, a grid was created around the co-crystallized ligand using the Glide grid module. It was intended to develop receptor grids that highlighted the ligand binding locations. Using the Glide tool, 30 ligands were docked into the binding site of protein structure 4ey7 in Schrodinger_Suites_2020_3 × 64. The docking procedure was carried out in Glide XP mode, which is designed to evaluate docking accuracy. After docking, the data was analyzed by visualizing the ligand postures using Schrodinger’s Maestro interface. This approach was used to investigate the ligand-Ache protein interactions under simulated conditions, which is consistent with previous studies [33–36].

#### 2.6.2. Structural interaction fingerprinting analysis.

To identify key residues involved in ligand binding, the study applied Structural Interaction Fingerprint (SIFt) analysis. This method provided a direct means of assessing interactions between ligands and receptors, holding substantial promise for drug discovery and design [37]. SIFt evaluates these interactions in three dimensions, simplifying the complexity of the ligand-protein binding characteristics into binary numbers, forming an interaction fingerprint. This condensed representation allows for organized analysis and display of data from ligand-receptor complexes, facilitating database mining [38]. When looking for substances with suitable binding modes inside the protein target in virtual chemical libraries, SIFt serves as a useful molecular filter. The classification of interactions into hydrophobic, H-bond donor, and H-bond acceptor properties aids in the identification of molecules with desired interaction patterns.

Using the SIFt panel in Schrodinger_Suites_2020_3 × 64, the research input receptor grids and ligands to produce interaction fingerprints for the protein-ligand complex [39]. The resulting fingerprints were visualized in Excel sheets, highlighting residues and their predominant interaction types. Colors denoted the interaction type, while “1” and “0” indicated presence and absence of interactions, respectively. The validation of docking results for 30 compounds was performed using fingerprint analysis, revealing diverse binding mechanisms, varied orientations, and different positions concerning the target protein across multiple poses.

#### 2.6.3. ADMET prediction studies.

Drug discovery and development in pharmacology and pharmaceutical research rely heavily on ADME (Absorption, Distribution, Metabolism, and Excretion) prediction studies. These studies use computer models and experimental data to forecast and assess how a prospective therapeutic ingredient would be absorbed, transported throughout the body, metabolized, and ultimately removed from the system. ADMET studies are significant in drug development because they examine a medicine’s physiological activity, notably its pharmacokinetic properties. Computational models have been created to save testing time while accurately assessing the ADMET profiles of specific drug combinations [40]. The compounds under investigation were evaluated for absorption, distribution, metabolism, excretion, and toxicity (ADMET) properties to identify their appropriateness as prospective therapeutic candidates. To acquire thorough information on the absorption and distribution profiles of the principal chemicals found via LC-MS analysis, the Swiss ADME online tool (available at https://www.swissadme.ch/index.php) was utilized [41]. Excretion properties were assessed using the online web tool pkCSM (https://biosig.unimelb.edu.au/pkcsm/prediction) [42].

#### 2.6.4. DFT Studies/MESP/HOMO/LUMO analysis.

The DFT calculations were performed with high accuracy with the help of Gaussian 09 software program (Revision E. 01) and the default parameters mentioned in the literature 23. The computations were performed by employing the B3LYP functional in combination with the SVP basis set and highlighted the potential of the methods to study electronic structures of atoms and molecules. The aim of this study is to consider several significant characteristics including the desired geometric features, FMO energies, the global and local reactivity descriptors, and MEP. These factors are vital for an in-depth understanding of the chemical properties and reactivity of the studied molecules, offering essential insights for theoretical and practical purposes. Checkpoint files produced during the simulations were examined using Gauss View 6, which facilitated detailed assessments of the electronic density distribution and potential energy landscapes

#### 2.6.5. Molecular Dynamic (MD) simulation.

The compounds CP7 Scandenin, CP10 Bergapten, and CP21 Dictyoquinazol C, which were identified and docked, underwent MD simulations to enhance the stability of their chemical structures bound to proteins. This involved a series of steps using software like Amber 20: assigning partial atomic charges to ligands with the antechamber module, integrating missing hydrogens and preparing the system for simulation with the Leap module. Force fields—ff14SB for the protein and generalized Amber force field (GAFF) for the ligand—were employed. The process included neutralizing the proton-containing protein, solvating the complex, and saving the solvated complex in PDB format along with preparing parameter and coordinate files. A stepwise minimization was carried out to eliminate any structural clashes within the system, focusing on protein, ligand, and the entire complex in successive phases. Each minimization stage involved specific steps and adjustments to ions and solvent systems. The system underwent heating and equilibration stages to stabilize at 300 K, followed by MD production for 100 ns under specific temperature and pressure conditions. Evaluations were performed using various parameters such as RMSD, RMSF, H-bond analysis, and distance analysis using the Amber 20 CPPTRAJ module. Additionally, we analyzed the dynamic stability and sampling methods by calculating the 2D Root Mean Square Deviation (RMSD) and Radius of Gyration (Rg) for the first 50 nanoseconds (ns) of the simulation. The RMSD values for ligands, protein binding sites, and apo-proteins were computed throughout the 100 ns simulation period, providing comprehensive insights into the system’s stability and behavior over time.

#### 2.6.6. Binding free energy calculations by MMPBSA/MMGBSA and energy decomposition analysis.

The study estimated binding free energies (BFE) by analyzing snapshots from molecular dynamics (MD) simulations. These energies were computed using the molecular mechanics MMPBSA/MMGBSA modules available in the AMBER 20 software [43]. The data was derived from the final 10 nanoseconds of 1000 snapshots taken from three distinct complex systems: CP7-Ache, CP10-Ache, and CP21-Ache. These calculations aimed to discern the energy difference between the inhibitor complex system (G_com_) and the free protein (G_ache_) according to equation (4).


$$\Delta {\text{G}}_{\text{bind}}=\;\Delta \text{H }-\text{ T}\Delta \text{S }=\;\Delta {\text{G}}_{\text{com}}\;-\;(\Delta {\text{G}}_{\text{cache}}+\;\Delta {\text{G}}_{\text{inh}})$$ (4)

We utilized data from the prior publication to obtain the computation factors for BFE (Binding Free Energy) [44–46]. These factors were defined as molecular mechanics energy (Emm) and solvation free energy (Gsol), calculated using the appropriate equations (5).


$${\text{E}}_{\text{MM}}=\;\Delta {\text{E}}_{\text{int}}\;+\;\Delta {\text{E}}_{vdW}\;+\;\Delta {\text{E}}_{\text{ele}}$$ (5)

The investigation focused on breaking down the molecular mechanics energy into specific components: van der Waals energies (E*vdW*), non-bonded electrostatic energies (E_ele_), and the solvation free energy (G_sol_). G_sol_, in particular, included both polar and nonpolar solvation energies (Eq 6).


$$\Delta {\text{G}}_{\text{sol}}=\;\Delta {\text{G}}_{\text{ele},\text{sol PB}(\text{GB})}\;+\;\Delta {\text{G}}_{\text{nonpol},\text{sol}}$$ (6)

In line with equation (6), the various factors contributing to the breakdown of energies within the inhibitor interaction were analyzed. This breakdown involved determining the decomposition parameters for van der Waals (G*vdW*), electrostatic (G_ele_), polar (G_ele_, _sol_), and nonpolar (G_nonpol_, _sol_) energies. These distinct contributions were investigated by dissecting the Binding Free Energy (BFE) into individual residual components. The analysis of these decomposition factors utilized the same snapshots that were employed for evaluating the Binding Free Energy.


$$\Delta {\text{G}}_{\text{inhibitor}-\text{residue}}=\;\Delta {\text{G}}_{vdW}+\;\Delta {\text{G}}_{\text{ele}}\;+\;\Delta {\text{G}}_{\text{ele},\text{sol}}\;+\;\Delta {\text{G}}_{\text{nonpol},\text{sol}}$$ (7)

Analyzing per-residue free energy decomposition provides a deeper understanding of how inhibitors bind and their selectivity, surpassing the insights gained from total binding free energy calculations. This method identifies specific residues that are critical to the binding energy, aiding in the design of inhibitors that more effectively target specific protein interactions or regions. By using MMGB/PBSA calculations, researchers can gain valuable insights into the molecular mechanisms of ligand binding. This understanding can lead to the development of more effective and targeted drugs.

### 2.7. Statistical analysis

The experiments were conducted in triplicate, with results presented as the mean ± standard deviation (S.D.). Data analysis was performed using GraphPad Prism, version 8. The assumptions of normality (assessed by Shapiro-Wilk test) and homogeneity of variance (assessed by Levene’s test) were confirmed. A one-way ANOVA was used, followed by Bonferroni’s multiple comparisons post hoc test. Significance was assigned to P values less than 0.05.

## 3. Results and discussion

### 3.1. Plant characterization

#### 3.1.1. Physicochemical analysis.

The physicochemical analysis of AJME indicates a plant material with relatively low moisture content (6%) and a notable presence of inorganic components, as seen in the total ash content (19%). The high levels of sulfated ash (48%) suggest the existence of compounds soluble in sulfuric acid, while the significant water-insoluble ash (38%) and alcohol-insoluble ash (17%) point towards specific mineral compositions and less soluble components. Additionally, the minimal quantities of water-soluble extractives (1.9%) and alcohol-soluble extractives (3.3%) hint at the limited presence of water- and alcohol-soluble compounds within this plant species (Table 1**).** Comparatively, other studies have investigated the phytochemical properties and biological activities of various *Acacia* species, revealing a range of therapeutic potentials. For instance, a study on *A. jacquemontii* demonstrated its significant antioxidant and hepatoprotective activities. The phenolic contents, including chlorogenic acid, P-coumaric acid, and kaempferol, were found in remarkable therapeutic ranges, contributing to its antioxidative and hepatoprotective effects [47].

**Table 1:** Physicochemical analysis and Total Bioactive contents of the studies extracts.

Physicochemical Parameters and bioactive contents	
Physicochemical Parameters	Percentage
Total ash content	19
Moisture content	6
Water-insoluble ash	38
Sulfated ash	48
Alcohol-insoluble ash	17
Alcohol-soluble extractives	3.3
Water-soluble extractives	1.9
**Quantitative phytochemical composition**	
**Total Bioactive contens**	**Quantity (mg/g)**
Total phenolic contents	25.8 ± 0.5
Total alkaloidal contents	42.5 ± 0.3
Total flavonoid contents	71.2 ± 0.4

#### 3.1.2. Phytochemical analysis.

The phytochemical analysis of AJME showcases significant quantities of various compounds within the plant material. It reveals a polyphenol content of approximately 25.8 mg/g with a margin of error of ±0.5, suggesting the presence of compounds like flavonoids and phenolic acids known for their antioxidant properties. Additionally, the total alkaloid content measures around 42.5 mg/g with a margin of error of ±0.3, indicating the existence of alkaline substances that might contribute to the plant’s pharmacological properties. Furthermore, the substantial presence of total flavonoids at approximately 71.2 mg/g with a margin of error of ±0.4 highlights the potential existence of compounds like flavones and flavonols, which often exhibit various health benefits due to their antioxidant and anti-inflammatory characteristics (Table 1). Similar high polyphenol content and antioxidant activities were observed in *A. jacquemontii* stem extracts, which demonstrated significant antioxidant and hepatoprotective effects [47]. Moreover, *A. nilotica* extracts also exhibited robust antioxidant activity, correlating with their high phenolic content [47].

Liquid Chromatography Mass Spectrometry (LCMS) Analysis: The LCMS analysis revealed a diverse spectrum of compounds (30 compounds) across various chemical classes (S1 Table). Among these compounds, ten belonged to the Pyrrolizidine class, with N-Methylcalystegine B2 standing as the identified compound. Pyrrolizidines are organic compounds characterized by their unique structure and are found in various natural sources. Alkaloids, another significant class, comprised five compounds. This class includes molecules like Homoarecoline, Loperamide, and Amitraz. Alkaloids are known for their diverse pharmacological activities and are commonly found in plants, often exhibiting potent biological effects.

The analysis identified D-Proline, an amino acid, showcasing its presence in the sample. Amino acids serve as the building blocks of proteins and play crucial roles in various physiological processes. Other classes represented include Piperidinecarboxamides, Formamidine, Tryptamine, Coumarins, Glycosides, and more. Each class offers unique chemical properties and functionalities, highlighting the complexity and richness of the analyzed sample. The presence of compounds across such varied chemical classes underscores the importance of comprehensive analytical techniques like LCMS in characterizing complex mixtures, providing insights into the diverse molecular composition of samples and their potential biological significance. A study has reported the isolation of new pyrrolizidine alkaloids and glycosides from *Anchusa strigosa*, showcasing the diversity of alkaloids present in plant sources [48].

#### 3.1.3. Antioxidant activity using DPPH assay and in-vitro ACHE.

The Table 2 presents the percentage inhibition of DPPH free radicals by ascorbic acid and AJME at various concentrations. Ascorbic acid shows increasing inhibition from 30.2% at 0.2 mg/ml to 75.3% at 1 mg/ml with an IC_50_ value of 0.52 mg/ml. In comparison, AJME shows lower inhibition, starting at 20.512% at 0.2 mg/ml and reaching 63.378% at 1 mg/ml, with an IC50 value of 0.69 mg/ml (Table 2**).**

**Table 2:** Percentage inhibition of DPPH analysis at different concentrations of AJME and IC_50_ values.

Conc. (mg/ml)	% inhib (ascorbic acid)	IC50 (mg/ml) – Ascorbic Acid	% inhib (AJME)	IC50 (mg/ml) – AJME
0.2	30.2	0.52	20.512	0.69
0.4	39.5		26.886	
0.6	49.5		38.176	
0.8	60.2		42.178	
1	75.3		63.378	

The percentage inhibition of AChE enzyme at concentration of 0.5 mg/mL and the IC50 values of methanol and dichloromethane extracts were also ascertained. Eserine was used as a positive control in the experiment (Table 3**).**

**Table 3:** AChE inhibition (%) and AChEIC_50_ AJME and AJD extracts.

Sample code	AChE inhibition (%)	AChE IC_50_ (μg/mL)
**AJME**	60.78 ± 0.11	200.81 ± 0.44
**AJD**	29.44 ± 0.55	** > 500
**Eserine**	92.29 ± 1.19	0.03 ± 0.001

Values are expressed as means ± SD of three replicates; ** IC_50_ value was higher than 500 μg/mL, AChE = acetylcholinesterase

AJME shows an AChE inhibition of approximately 60.78%, with an IC50 of about 200.81 μg/mL. AJD demonstrates an AChE inhibition of roughly 29.44%. However, its IC50 value is greater than 500 μg/mL. Eserine exhibits the highest AChE inhibition at around 92.29%, with an impressively low IC_50_ value of 0.03 μg/mL. Comparing these results, Eserine appears to be the most effective in inhibiting AChE, displaying both a high percentage of inhibition and an incredibly low IC_50_ value, indicating strong potency at very low concentrations. AJME also shows decent inhibition and moderate potency, while AJD exhibits a lower percentage of inhibition and a high IC_50_ value, suggesting it might be less effective against AChE compared to the other compounds tested. A combination of *A. catechu* and *Scutellaria baicalensis* extracts has been shown to alleviate joint discomfort and reduce inflammation, which indicates some level of bioactivity, including potential AChE inhibition effects. However, the direct measurement of AChE inhibition in these studies was not performed [49].

### 3.2. Anti-parkinsonian activity (behavioral analysis)

#### 3.2.1. Narrow beam walk test.

**Fig 1:**
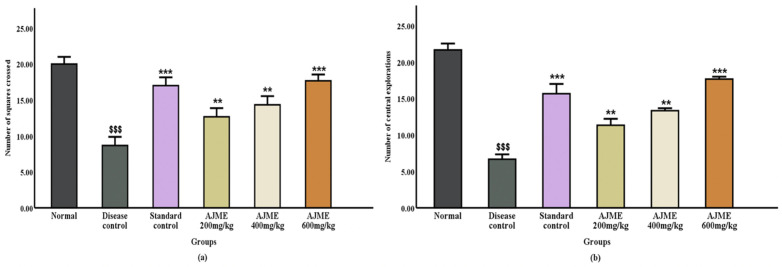
The impact of AJME on the time taken (in seconds) during the narrow beam walk test was assessed. Results are expressed as mean ± SEM (n = 6). Significant differences were noted: ^$$$^P < 0.001 compared to the normal control group. ^**^P < 0.01 and ^***^P < 0.001 compared to the disease control group.

The study evaluated balance by counting padded errors and assessed motor function and coordination on the 21st day post-dosage using the latency period in a narrow beam test. Significant differences (P < 0.001) were observed in delay time and the frequency of foot errors when comparing the haloperidol-treated group to the AJME-treated group (Fig 1).

#### 3.2.2. Catalepsy test.

**Fig 2:**
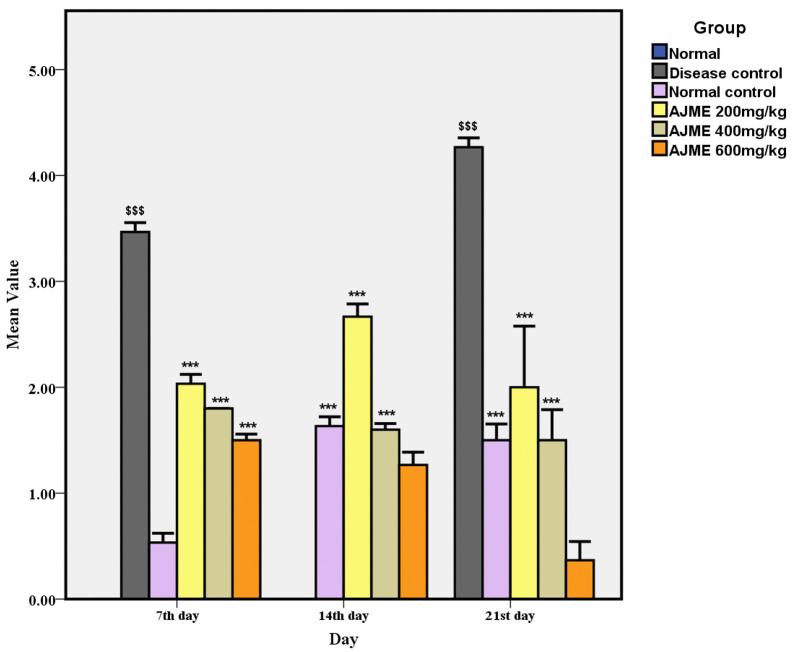
The impact of AJME on the assessment of cataleptic scores is depicted. Results are displayed as mean ± SEM (n = 6), with significance denoted as $ for P < 0.05, ^$$^ for P < 0.01, and ^$$$^ for P < 0.001 concerning comparisons with the normal control group. Moreover, asterisks (*) represent significance levels: ^*^ for P < 0.05, ^**^ for P < 0.01, and ^***^ for P < 0.001 when compared to the disease control group.

After drug administration on each test day, the length of cataleptic reactions was noted for every animal at 30, 60, 90, and 120 minutes. The test was conducted on Days 7, 14, and 21 after daily administration. The results of the catalepsy test are shown in Fig 2. Compared to the control group, the haloperidol (HAL) group experienced a significant increase (P < 0.001) in the amount of time spent in a cataleptic condition on all testing days. In contrast to the HAL-only group, however, these cataleptic effects were reversed by oral administration of typical anti-parkinsonian medications, levodopa and carbidopa, in a dose- and time-dependent manner (P < 0.001). Furthermore, groups treated with AJME exhibited a dose-dependent decrease in cataleptic reactions. When compared to the illness group, the 600 mg/kg dose dramatically reduced catalepsy (P < 0.001), with the lowest scores seen on the 21st day.

AJME’s ability to reduce cataleptic reactions in a dose-dependent manner is comparable to the effects observed with traditional anti-Parkinsonian medications like levodopa and carbidopa. Similarly, a study evaluating the antiparkinsonian effects of a hydroalcoholic extract mixture of Camellia sinensis, Asparagus racemosus, and Mucuna pruriens showed significant reductions in catalepsy, suggesting that these plant extracts might be effective in alleviating PD symptoms [50].

#### 3.2.3. Hole board test.

**Fig 3:**
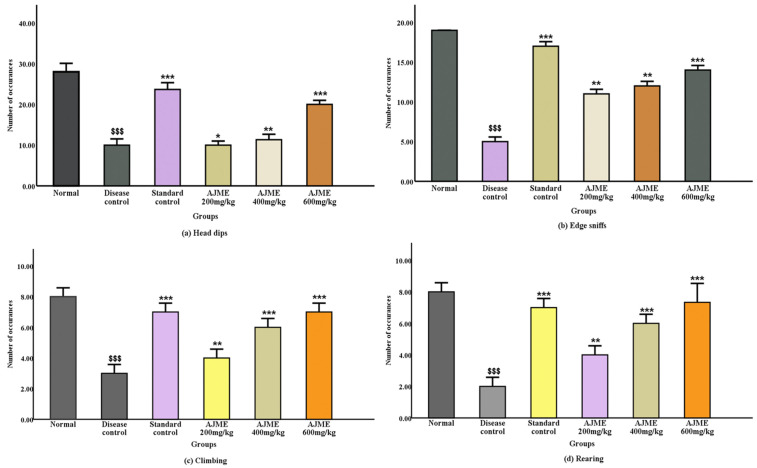
The impact of AJME on (a)(b) focused exploratory activities and (c)(d) vertical exploratory activities in the hole board test was evaluated. Results are displayed as mean ± SEM (n = 6). Significant differences were observed: ^$$$^ indicating P < 0.001 compared to the normal control group, while ^*^ denoted P < 0.05, ^**^ indicated P < 0.01, and ^***^ signified P < 0.01 compared to the disease control group.

Each experimental animal group’s distinct exploring habits, both vertical and horizontal, and any potential anti-anxiety effects were evaluated using the hole board test. The quantity of rats investigating holes functioned as a measure of curiosity. A post-analysis showed that the group receiving haloperidol alone had significantly less hole probing (P < 0.001). As seen in Figs 3a–3d, there was a significant decrease in concentrated behaviors including edge sniffing and head dipping in the illness group (P < 0.001). All treatment groups demonstrated a rise in their inquisitive behavior. When it came to climbing and rearing activities, the AJME dose groups of 200 mg/kg and 400 mg/kg exhibited considerable recovery (P < 0.01), while the 600 mg/kg dose group showed significant improvement (P < 0.001). The plant extract’s dose-dependent activity raises the possibility that it can stop dopaminergic loss (Fig 3**).**

#### 3.2.4. Open-field test.

**Fig 4:**
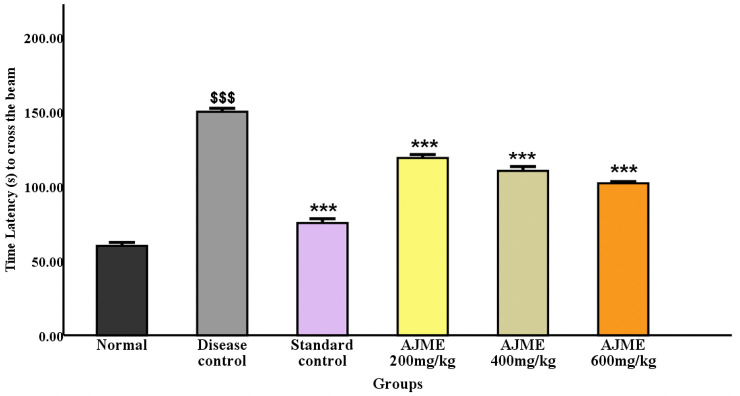
The impact of AJME on (a)(b) focused exploratory activities and (c)(d) vertical exploratory activities in the hole board test was evaluated. Results are displayed as mean ± SEM (n = 6). Significant differences were observed: ^$$$^ indicating P < 0.001 compared to the normal control group, while ^*^ denoted P < 0.05, ^**^ indicated P < 0.01, and ^***^ signified P < 0.01 compared to the disease control group.

**3.2.4.1. Number of squares crossed and Number of central explorations**: The study investigated the effects of AJME, a plant extract, on rat behavior, focusing on locomotor activity and exploratory behavior. The results, depicted in Figs 1a, 1b, showed significant influences of the treatment groups on the number of squares traversed and exploration in both central and peripheral areas (P < 0.001). Post-test analysis indicated that doses of 100 mg/kg and 300 mg/kg led to notable increases (P < 0.01), with the most significant improvement in central area explorations (P < 0.01). Furthermore, rats treated with the plant extract exhibited a dose-dependent enhancement in the number of squares traversed (P < 0.05, P < 0.01, and P < 0.001 for increasing doses), reflecting increased locomotor activity. This suggests a possible anxiolytic effect of AJME. In contrast, the disease control group showed a significant decrease (P < 0.001) in both central and horizontal explorations, indicating higher anxiety levels. Rats treated with AJME displayed increased exploratory behavior and locomotor activity, implying reduced anxiety compared to the control group (Fig 4**).**

#### 3.2.5. Y-Maze test.

**Fig 5:**
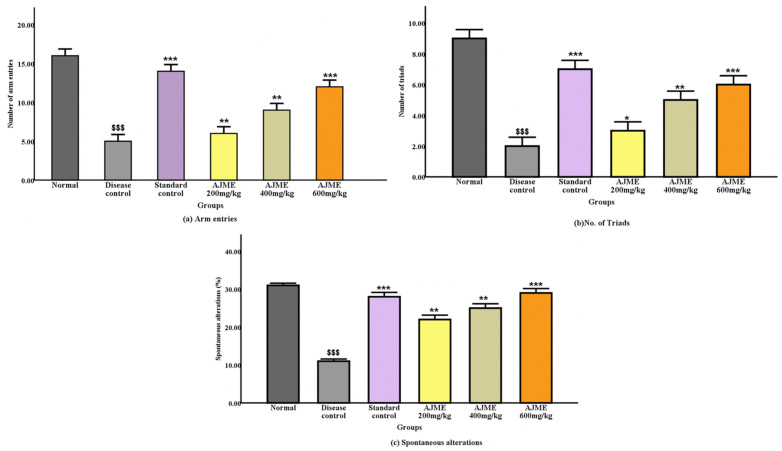
Impact of AJME on (a) arm entries, (b) number of Triads, and (c) spontaneous alterations (%) in the Y-maze test. The data is depicted as mean±SEM (n = 6). Results indicate significance at P < 0.001 compared to the normal control group. Moreover, asterisks denote significance levels (*P < 0.05, **P < 0.01, and ***P < 0.001) in comparison to the disease control group.

The effect of AJME on working or short-term memory was evaluated using the Y-maze test for spontaneous alternation behavior. When compared to the normal control group, the haloperidol therapy resulted in a significant (P < 0.001) decrease in spontaneous alternation behavior. This decline was significantly reversed in a dose-dependent fashion when AJME was given at dosages of 200, 400, and 600 mg/kg (P < 0.05, P < 0.01, and P < 0.001). Haloperidol was similarly found to reduce the number of arm entries in the disease control group; however, in the therapeutic groups, memory loss significantly improved (P < 0.001). The percentage of spontaneous alternations increased significantly (P < 0.001) in the standard and 600 mg/kg dose groups. Fig 5a–5c displays the results (Fig 5).

#### 3.2.6. Swim test.

Following the administration of AJME and haloperidol at varying doses in the Parkinson’s disease model, the degree of symptoms was assessed using swimming scores, as presented in Table 4. Increased exploring activities were seen in all treated groups. The 200 mg/kg and 400 mg/kg AJME groups had a substantial improvement (P < 0.01) in their climbing and rearing activities, whereas the 600 mg/kg AJME group demonstrated a considerable improvement (P < 0.001). The plant extract’s dose-dependent action raises the possibility of defense against dopamine deficiency. Furthermore, a noteworthy reduction in the length of immobility (P < 0.001) was noted in relation to the group that was not affected by the illness. In comparison to the disease control group, the standard therapy group also showed a significant improvement in swimming performance (P < 0.001) and a significant dose-related decrease (P < 0.001) in immobility duration. The group receiving the conventional treatment also showed a significant improvement in their ability to swim (P < 0.001).

**Table 4:** Effect of *AJME* on forced swim test.

Groups	Dose (mg/kg)	Swimming (sec)	Climbing (sec)	Immobility (sec)
Normal control	—	147 ± 3.69	29.50 ± 3.17	76.51 ± 5.05
Disease control (Haloperidol)	1	100.2 ± 5.40 ^$$$^	18 ± 3.5 ^$^	120 ± 7.5 ^$$^
Standard (Levodopa+carbidopa)	100/25	145.3 ± 4.20 ^***^	27.2 ± 3.32 ^***^	80.3 ± 5.34 ^***^
*A. jacquemontii*	200	125.09 ± 4.2^*^	26.09 ± 3.4^**^	110.23 ± 6.3^*^
	400	140.65 ± 3^**^	19.98 ± 4.6^**^	95 ± 10.7^**^
	600	143.09 ± 3 ^***^	20.5 ± 4^***^	86.5 ± 4^***^

Data displayed as mean ± SEM (n = 6). ^$^ Signifies P-values < 0.05, and ^$$$^ Signifies P-values < 0.001 in comparison to the normal control group. ^*^ Indicates P-values < 0.05, ^**^ Signifies P-values < 0.01, and ^***^ Represents P-values < 0.001 in comparison to the disease control group.

The open-field test results from the study show that AJME increased locomotor activity and reduced anxiety in treated rats. These findings align with those from a study on the neuroprotective activities of medicinal plants used by the Pikuni-Blackfeet tribe, which found that extracts from plants like *Allium sativum* and *Amelanchier arborea* could protect against neurotoxicity and improve locomotor functions in PD models [51]

### 3.3. Oxidative stress parameters in the brain

#### 3.3.1. Superoxide Dismutase (SOD) levels.

After inducing parkinsonism with haloperidol for 21 days, there was a significant decrease in brain tissue SOD levels (P < 0.001). Conversely, groups treated with AJME at a dosage of 600 mg/kg showed a significant increase in SOD levels (P < 0.001) after the same period (Table 5). While levodopa and carbidopa also raised SOD levels in haloperidol-treated rats, this improvement was not statistically significant compared to the increase observed in rats treated with the extract.

**Table 5:** Estimation of SOD, CAT, and GSH levels in brain homogenates.

Groups	Dose	SOD (μg/mg of protein)	CAT (μmol/min/mg of protein)	GSH (μg/mg of protein)
**Normal control**	—	2.2 ± 0.02	30.2 ± 0.2	12.2 ± 0.1
**Disease control**	1mg/kg	1.32 ± 0.02^$$$^	22.6 ± 0.3^$$$^	8.37 ± 0.1^$$$^
**Standard**	100/25 mg/kg	2.17 ± 0.01***	28.6 ± 0.2***	12.66 ± 0.1**
***A. jacquemontii***	200 mg/kg	1.67 ± 0.03*	27.0 ± 0.3*	8.78 ± 0.7**
	400 mg/kg	1.95 ± 0.02**	26.7 ± 0.3**	12.44 ± 0.5**
	600 mg/kg	2.04 ± 0.01***	28.1 ± 0.4***	13.17 ± 0.5***

Data displayed as mean ± SEM (n = 6). ^$$$^ indicates significance at P < 0.001 compared to the normal control group. Additionally, ^*^ denotes significance at P < 0.05, ^**^ at P < 0.01, and ^***^ at P < 0.001 compared to the disease control group.

#### 3.3.2. Catalase (CAT) levels.

Table 5 compares the haloperidol-treated group to the normal control group over a 21-day period and shows a substantial decrease in catalase levels (P < 0.001). The change in CAT levels brought about by haloperidol was successfully undone by the conventional treatment group. However, when AJME’s aqueous methanolic extract was given with haloperidol, a dose-dependent and significant improvement was seen in all treatment groups—200 mg/kg (P < 0.001), 400 mg/kg (P < 0.001), and 600 mg/kg (P < 0.001).

#### 3.3.3. Reduced Glutathione (GSH) levels.

Table 5 indicates that the disease control group experienced a significant reduction (P < 0.001) in GSH levels in brain tissue homogenates following haloperidol administration. The group treated with levodopa and carbidopa, in addition to haloperidol, exhibited GSH levels that were nearly normal. The treatment groups receiving 200 mg/kg and 400 mg/kg doses of AJME showed moderately significant improvements (P < 0.01), similar to the GSH level restoration observed in the standard group (P < 0.05). Notably, the group treated with 600 mg/kg of AJME demonstrated a highly significant recovery of GSH level (P < 0.001).

#### 3.3.4. Malondialdehyde (MDA) levels.

Rats treated with haloperidol exhibited significantly higher MDA levels (P < 0.001) compared to the normal control group. Concurrent administration of the AJME aqueous methanolic extract significantly decreased MDA levels across all treatment doses (P < 0.05). However, after 21 days of treatment with levodopa and carbidopa, the standard treatment group showed a significant decline in MDA levels, nearly matching those of the normal control group (Table 6).

**Table 6:** Estimation of MDA and nitrite levels in brain homogenates.

Groups	Dose	MDA (nmol/mg of protein)	Nitrite (ug/mg of protein)
**Normal control**	—	730 ± 2.5	2.0 ± 0.2
**Disease control**	1mg/kg	740 ± 1.7^$$$^	2.2 ± 0.2^$$$^
**Standard**	100/25 mg/kg	820 ± 1.6**	2.43 ± 0.3**
***A. jacquemontii***	200 mg/kg	790 ± 1.2**	3.0 ± 0.2***
	400 mg/kg	765 ± 1.2**	3.2 ± 0.2***
	600 mg/kg	750 ± 1.8***	2.5 ± 0.2***

Data displayed as mean ± SEM (n = 6). ^$$$^ indicates significance at P < 0.001 compared to the normal control group. Additionally, ^*^ denotes significance at P < 0.05, ^**^ at P < 0.01, and ^***^ at P < 0.001 compared to the disease control group.

#### 3.3.5. Nitrite levels.

Table 6 demonstrates that nitrite levels were significantly reduced at 200 mg/kg of AJME (P < 0.05) and even more significantly at 400 mg/kg and 600 mg/kg doses (P < 0.001). The standard therapy group also showed a significant reduction in nitrite levels (P < 0.001). Conversely, nitrite levels in the disease control group remained elevated with haloperidol treatment.

The significant improvement in oxidative stress markers such as SOD, CAT, and GSH levels in the brain following AJME treatment highlights its potential antioxidant properties. This is corroborated by findings from other studies where herbal extracts showed neuroprotective and antioxidant effects. For instance, *Bacopa monnieri* has been reported to improve emotional function and oxidative stress parameters in PD patients, though further clinical trials are needed [52].

### 3.4. Neurotransmitter levels estimation in the brain

#### 3.4.1. Dopamine, noradrenaline and serotonin levels.

**Fig 6:**
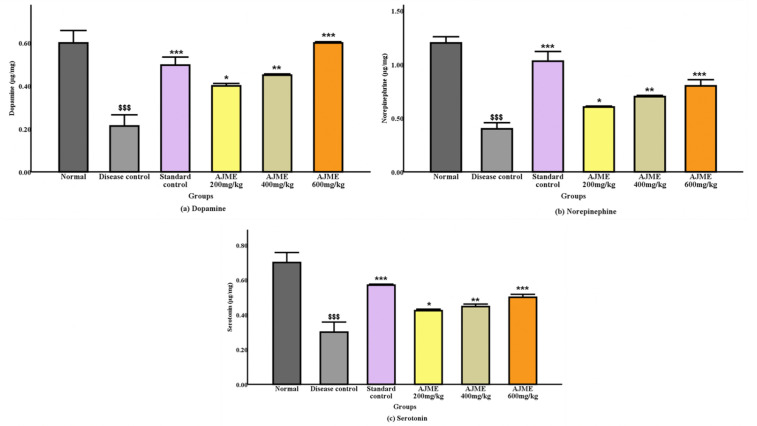
Impact of AJME on (a) dopamine, (b) nor-adrenaline, and (c) serotonin levels in brain homogenates was evaluated. The data is represented as mean ± S.E.M (n = 6). Significant differences were observed (P < 0.001) when compared with the normal control group. Moreover, asterisks denote significance levels: ^*^ for P < 0.05, ^**^ for P < 0.01, and ^***^ for P < 0.001 in comparison with the disease control group.

Serotonin, noradrenaline, and dopamine levels in brain tissue homogenate were measured. Memory recovery was significantly (P < 0.001) improved in the therapy groups. Naturally occurring changes significantly increased (P < 0.001) in the 600 mg/kg and standard dosing groups. Neurotransmitter levels were significantly increased in both the AJME-treated and standard groups; this effect was similar to that of haloperidol therapy. As seen in Figs 6a,6b, all treatment groups did, however, show a dose-related improvement in dopamine and noradrenaline levels (P < 0.05, P < 0.01) at 200 mg/kg and 400 mg/kg, with a significant (P < 0.001) rise at the highest dose of 600 mg/kg.

The levodopa + carbidopa (standard)-treated group showed a highly significant (P < 0.001) improvement in serotonin levels compared to the disease control group, which had lower serotonin levels (Fig 6c). Dyskinesia and mood disorders are associated with decreased serotonin levels in Parkinson’s disease patients. Serotonin levels were recovered in a dose-dependent way at all AJME dosage levels, with a notable increase (P < 0.001) at the highest dose of 600 mg/kg.

#### 3.4.2. Acetyl Cholinesterase (AChE) activity.

**Fig 7:**
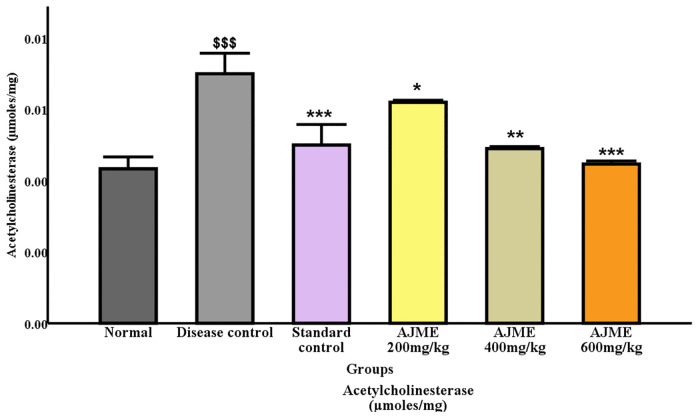
Results are expressed as mean ± S.E.M. (n = 6). Significant differences were observed (P < 0.001) compared to the normal control group. Additionally, distinctions were noted with *P < 0.05, **P < 0.01, and ***P < 0.001 compared to the disease control group.

In the disease control group, the AChE levels significantly increased (P < 0.001). On the other hand, AChE levels decreased in the group receiving conventional treatment (P < 0.001). The 600 mg/kg dosing group of AJME showed a highly substantial (P < 0.001) drop in AChE levels. Simultaneously, improvements were also observed at dose levels of 200 mg/kg and 400 mg/kg (P < 0.05) (Fig 7**).**

While the current study suggests AJME as a potential treatment for PD, it is essential to compare its efficacy with well-established treatments. A study on the use of Mucuna pruriens as an adjunct therapy to levodopa in advanced PD found that it could provide a clinical motor effect similar to conventional levodopa, with the added benefit of reducing “off” time without exacerbating dyskinesias [53].

### 3.5. Histopathology of brain tissue

**Fig 8:**
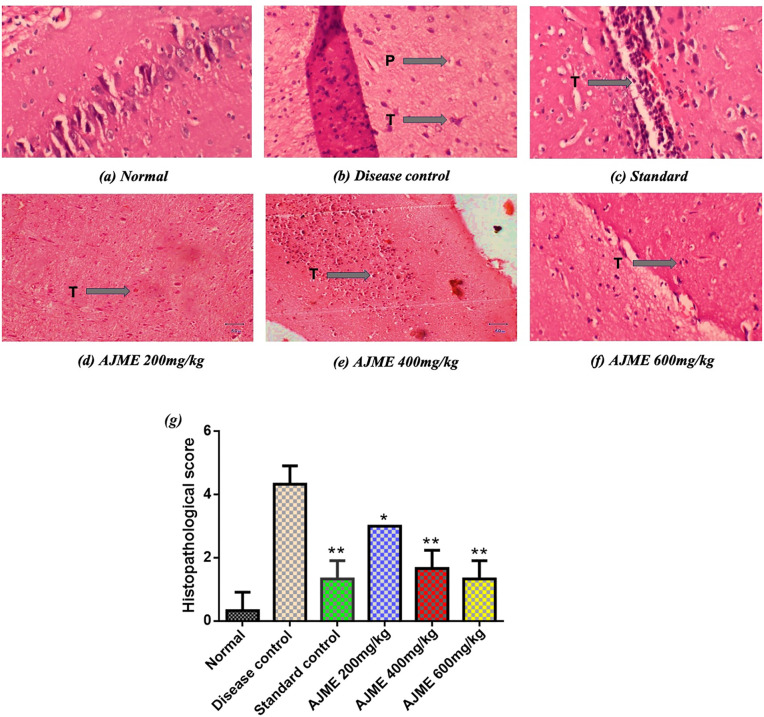
The histopathological examination of brain tissue reveals significant insights into the effects of various treatments on neurodegenerative conditions. In (a), the normal brain tissue displays typical architecture with no pathological features. In contrast, Fig (b) illustrates the disease control group, where the presence of plaques (P) and neurofibrillary tangles (T) highlights the progression of the disease. The standard treatment group, depicted in (c), shows a notable reduction in neurofibrillary tangles (T), indicating the efficacy of the treatment. (d), (e), and (f) present the effects of methanolic extract of *A. jacquemontii* (AJME) at different dosages. In (d), treatment with a 200 mg/kg dose of AJME results in a visible reduction of neurofibrillary tangles (T). This effect is further enhanced in Fig (e), where a 400 mg/kg dose of AJME leads to a more pronounced decrease in neurofibrillary tangles (T). (f) demonstrates that a 600 mg/kg dose of AJME achieves significant improvement, with minimal neurofibrillary tangles (T) observed, underscoring the potential therapeutic benefits of AJME in treating neurodegenerative conditions. (g) quantification of histopathological slides. Statistical differences were denoted by *P < 0.05, and **P < 0.01 compared to the disease control group.

Fig 8 shows the histological alterations in brain samples from various treatment groups. Brain samples from the normal control group showed no pathological alterations and an intact histological structure under a light microscope at 40 × magnification. On the other hand, there was modest bleeding, mild vascular congestion, and neuronal degeneration in the disease control group. Additionally, the brain tissues of the group that received haloperidol treatment showed signs of neurofibrillary tangles and plaques, which were significantly lowered as compared to disease control (*P* < 0.01). There was a noticeable improvement in histological changes (*P* < 0.05, < 0.01, and <0.01) in the groups which received 200, 400, and 600 mg/kg of AJME treatment. A study on Pikuni-Blackfeet traditional medicine identified several medicinal plants with neuroprotective activities, such as Allium sativum and Amelanchier arborea. These plants exhibited neuroprotective activity against PD-related neurotoxicity, similar to the findings with AJME, which showed improvements in histopathological features [51]. Another study evaluated a combination of hydroalcoholic extracts from Camellia sinensis, Asparagus racemosus, and Mucuna pruriens for their antiparkinsonian activity. The study found significant reductions in neurodegenerative markers and improvements in brain histology, which aligns with the improvements seen in the AJME-treated groups [50].

### 3.6. *In silico* studies

#### 3.6.1. Molecular docking studies for Acetyl cholinesterase (AChE) Inhibition.

To elucidate the distinctions in docking scores derived from different binding interactions, molecular docking studies were performed on Eserine (standard) and various natural compounds identified through LC-MS from the extract of AJME, as tabulated in S1 Table. The AChE docking scores for these compounds ranged from −4.262 to −10.016 kcal/mol. Eserine, denoted as PHY in the study, displayed a docking score of −9.512 kcal/mol. Among the compounds analyzed, CP1 showed the highest binding affinity towards AChE with a docking score of −10.016 kcal/mol, suggesting a superior interaction profile, as outlined in S1 Table. Comparatively, CP21 demonstrated a docking score of −9.091 kcal/mol, closely aligning with the performance of the standard compound, Eserine. CP10 and CP7 recorded docking scores of −6.406 and −8.433 kcal/mol, respectively, indicating varying degrees of affinity. CP21 was highlighted as the optimal ligand, not only for its comparable docking score to Eserine but also due to its structural congruence with the standard and its advantageous synthetic accessibility. These findings are further elaborated in the context of their binding modes and ADMET profiles.

**Fig 9:**
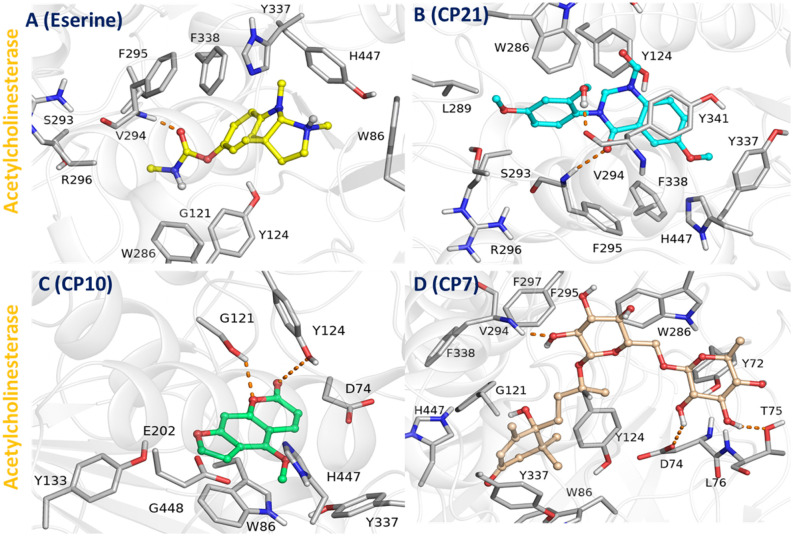
Docking-generated complex involving enzyme AChE with standard inhibitor (Eserine) and tested compounds (CP21, CP10 and CP7). Complex of AChE with Eserine **(A)**, AChE-CP21 **(B)**, AChE-CP10 (C) and AChE-CP7 **(D)**.

To explore the variations in docking scores due to distinct interaction mechanisms, optimal docking configurations for each compound were recorded and visually represented (Fig 9**).** All ligands, alongside benchmark inhibitors, are located within the identical binding pocket of AChE. The graphical depiction in Fig 9 reveals that AChE-ligand complexes exhibit shared interacting residues, namely D72, Y70, Y121, W279, F290, F288, I287, and Y334. In contrast, the interaction profile of the AChE-Eserine complex is characterized by engagement with a unique set of residues, including S293, R296, W286, G121, Y124, W86, H447, Y337, F338, and F295, as depicted in (Fig 9 **Image A).** Eserine forms a critical hydrogen bond through the oxygen extending from its structure to the amino group of residues V294 situated in the Mid Gorge region of AChE. Additionally, it facilitates hydrophobic interactions with aromatic residues such as F295, F338, W286, Y124, W86, H447, and Y337. The proximity of the aromatic rings of residues H337, H447, and W86 to the pyrrolidine rings of Eserine enhances these interactions, contributing to the specificity and stability of the binding. The graphical representation discloses that the AChE-ligand complexes share common interacting residues D72, Y70, Y121, W279, F290, F288, I287, and Y334 as demonstrated in (Fig 9**).**

In contrast, the AChE-CP21 complex interacts with a distinct set of residues, including W286, Y124, L289, S293, F295, H447, Y341, V294, F338, and R296, as illustrated in (Fig 9 **Image B)**. The CP21 forms two crucial hydrogen bonds: one with residue Y341 and another with V294, similar to the interaction observed with Eserine. The oxygen atom from the diazine ring of CP21 forms a hydrogen bond with the amino group of V294 located in the Mid Gorge region, mirroring the Eserine interaction. Another hydrogen bond occurs between the methyl-hydroxyl group attached to the benzene ring of CP21 and the backbone oxygen of residue Y341 in the gorge region of AChE. Hydrophobic interactions further underscore the efficacy of CP21, particularly due to the presence of aromatic residues such as W286, Y124, F295, and Y341, which cover 40% of the total surface area of the gorge region. The parallel alignment of Y341’s benzene ring to CP21’s benzene structure, along with the proximity of Y124’s benzene ring to the diazine ring of CP21, facilitates strong π-π interactions. Additionally, aliphatic chains from residues L289, S293, and R296 engage in van der Waals interactions with the benzene ring linked to the diazine ring’s amino group, enhancing the binding specificity and stability within AChE’s active site.

To explore the variations in docking scores stemming from distinct binding dynamics, the AChE-CP10 complex was thoroughly examined and visually detailed in (Fig 9 **Image C).** The complex prominently involves key residues D74, H447, Y337, W86, G448, E202, and Y133. CP10 establishes robust binding through crucial hydrogen bonds with residues G121 and Y124, while simultaneously enhancing hydrophobic interactions via residues W86, Y133, E202, and H447, spanning both the catalytic active site (CAS) and peripheral anionic site (PAS) of AChE, thereby augmenting its specificity and stability. The oxygen atom attached to the pyran ring of CP10 forms a hydrogen bond with the hydroxyl group extended from residue Y124 located in the CAS region. Additionally, the oxygen present within the pyran ring of CP10 engages in a hydrogen bond with the hydroxyl group of the aliphatic chain from residue G121, also within the CAS region. Aromatic residues W86 and Y337 in the PAS region, along with H447 in the CAS region, participate in π-π interactions with the furan and benzene rings of CP10, enhancing the ligand’s alignment and interaction within the binding pocket. Furthermore, the aliphatic chain from residue G448 contributes to the binding dynamics through van der Waals interactions with the furan ring of CP10, reinforcing the molecular conformation and stability within the active site of AChE.

To elucidate the variances in docking scores resultant from diverse interaction modalities, the optimal docking configurations for the AChE-CP7 complex were documented and depicted graphically (Fig 9 **Image D).** This complex engages various residues including F297, V294, F295, F338, G121, H447, Y337, W86, Y124, D74, L76, T75, Y72, and W286. CP7 forms three significant hydrogen bonds with residues D74, T75, and V294, enhancing its binding stability. Specifically, the Methoxytetrahydro-2H-pyran-3-4-5-triol ring of CP7, situated in the PAS region, forms two hydrogen bonds: one between the 3R-hydroxyl group and the oxygen extended from residue D74, and another between the 4R-hydroxyl group and the oxygen extended from the aliphatic chain of residue T75. Additionally, another methoxy-tetrahydro-pyran-triol ring centered in CP7 creates a hydrogen bond between its 3R-hydroxyl group and the amino group of residue V294. CP7 is located between the PAS and the mid-gorge region, with its benzene ring extended into the CAS region where residues G121 and Y124 participate in hydrophobic π-π interactions. Furthermore, aromatic residues F295 and W286 in the mid-gorge region engage in π-π interactions with the centered pyran ring of CP7. Similarly, residue Y72 of the PAS region also establishes π-π interactions with the pyran ring extended into the PAS region of AChE.

The aliphatic chains of PAS region residues D74, L75, and L76 also contribute to the stability of CP7 through van der Waals interactions with the pyran ring, thereby underlining the complexity and specificity of the interactions within the AChE binding site. A research focused on pharmacophore-based virtual screening and identified novel AChE inhibitors with high binding affinities and significant inhibitory effects. The inhibitors showed strong interaction within the active site of AChE, similar to standard inhibitors like Tacrine and Galantamine [54]. Fang et al. (2014) investigated genistein derivatives and their AChE inhibition mechanisms, identifying crucial interactions with residues like Tyr124, Glu292, and Phe338. The study found that electrostatic contributions significantly impacted binding affinities [55].

#### 3.6.2. Structural interaction fingerprinting analysis.

**Fig 10:**
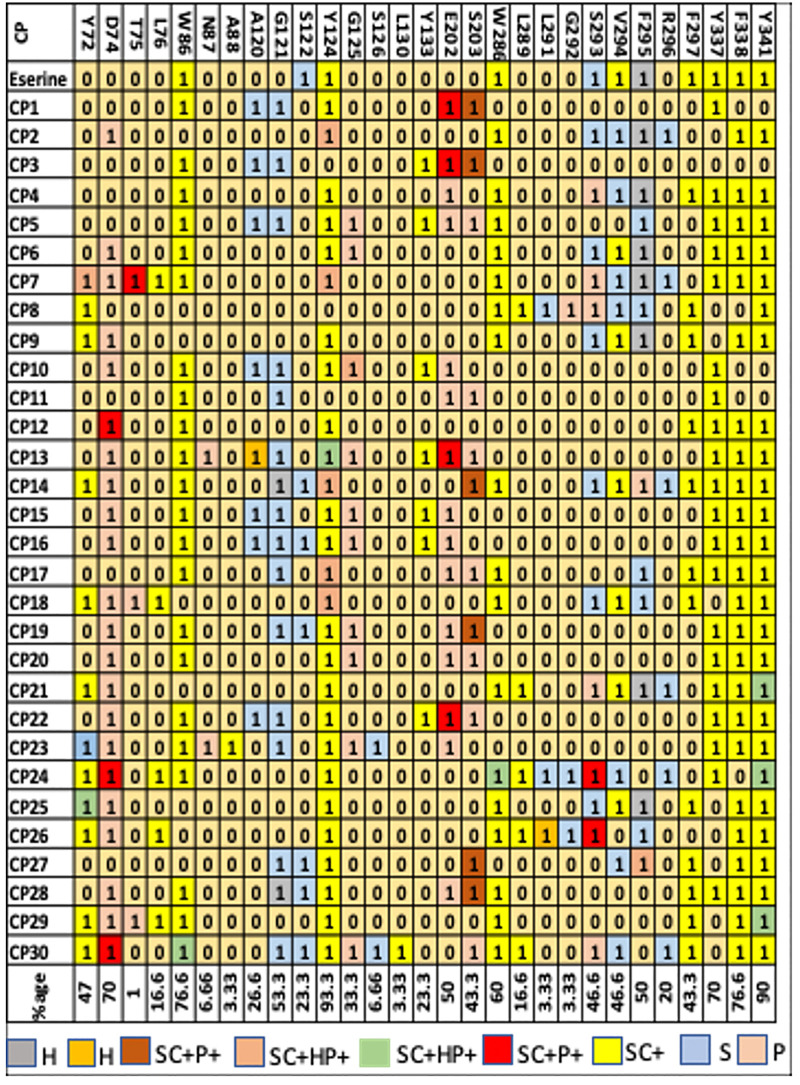
SIFt Analysis: Protein-ligand interaction fingerprints for modelling compounds and VS-hits within a 4.0Å radius of interacting residues. Residue presence indicated as 1, absence as 0, and colored by hydrophobic, hydrogen bond donor, and acceptor properties.

One of the earliest and most well-known fingerprints is the structural interaction fingerprint (SIFt). As illustrated in Fig 10, SIFt is a binary fingerprint composed of a seven-bit vector for each amino acid, representing the interaction pattern between the residue and the ligand. This fingerprint can involve main chain or side chain atoms, encompass various types of interactions (including hydrophobic and aromatic), and indicate whether the residue functions as a hydrogen bond donor or acceptor. Residues present in the binding cavity of AChE-Eserine are S293, R296, W286, G121, Y124, W86, H447, Y337, F338, and F295 as shown in (Fig 9 Image A). These are the same residues that present in the binding cavity of AChE-CP21 as shown in Fig 9 ImageB. Residues present in JACK-CP21 complex are W86, Y124, L289, S293, F295, Y341, V294, F338, and R296. These residues L76, T75, Y72, D74, W86, N87, G121, S122, Y124, G125, S126, L130, Y133, E202, S203, W286, L289, L291, S293, F295, V294, R296, F297, Y337, F338 and Y341 are also present in fingerprinting analysis as shown in Fig 10. Hydrogen bond acceptor interaction present in L289 with ligand CP26, can be seen in orange color (H-bond accept) and grey color (H-bond donor) in the fingerprint analysis (Fig 10). Almost all ligands are involved in conserved interaction with Y124, E202, S203, V294 and F295 are the most crucial residues in the binding cavity of AChE because they are essential for residual interaction. Like CP21 mostly compounds have hydrogen bond interaction with residue D74, G121, V294, F295, Y124 and Y341 have pink color (Donor H-bond, hydrophobic and side chain interaction) and green (H-bond acceptor, side chain and hydrophobic interaction) as shown in Fig 10. Hydrogen bond donor, polar and side chain interaction between residues S223 and ligands CP1, CP3, CP14, CP19, CP27 and CP28 as shown in brown color in the fingerprint analysis (Fig 10**).**

All the residues present in binding cavity shown interaction in fingerprinting analysis but these residues Y72, L76, N87, S122, G125, S203, L130, S126 and L291 are not present in the binding cavity of AChE but involved in making hydrogen bond acceptor and donor interaction with ligand CP7, CP25, CP13, CP26 as shown in grey color as hydrogen bond donor, brown color for side chain, polar and H-bond donor interaction then hydrogen bond acceptor with side chain and polar interaction show in red color in the table of fingerprinting. Hydrogen bond donor and side chain interaction present between GLN288 with ligand CP21, CP24 and CP84, as shown in dark green color in the fingerprint analysis table. Many residues Y72, W86 Y124, Y133, W286 V295, F297, Y337, F338 and Y341 have side chain, hydrophobic interaction shown in yellow color as given in the fingerprinting table. Residues A120, G121, S122 and S293 have contact and polar interaction shown in skyblue and peach color respectively, as given in fingerprinting table. Fingerprinting graph can see seen in S1 Fig. Fingerprinting analysis conclude that all the ligands not only are similar in shape but also have similar electrostatic pattern or interaction. A study by Fang et al. (2014) on the inhibition of AChE by genistein derivatives provides a relevant comparison. The study identified critical residues such as Tyr124, Glu292, and Phe338, which are involved in significant hydrogen bonding and electrostatic interactions, similar to the residues identified in the current fingerprinting analysis [55].

#### 3.6.3. ADME prediction studies.

The findings of ADME prediction studies are presented in the S2 Table, which provides comprehensive information on various aspects of the compounds investigated. These include ADME characteristics (absorption, distribution, metabolism, and excretion), physicochemical parameters, pharmacological toxicity, mutagenesis profile, and synthetic accessibility. Synthetic accessibility values in the range of 1 indicate compounds that can be readily synthesized, while values closer to 10 signify compounds that pose significant challenges in terms of synthesis [56]. Among the identified compounds, one with the highest synthetic accessibility has a value of 6.81 and corresponds to CP7. Conversely, the compound with the lowest synthetic accessibility has a value of 1.54, attributed to CP3. To evaluate the *in silico* ADMET properties of these molecules, the pkCSM online tool (https://biosig.lab.uq.edu.au/pkcsm/prediction) was employed. When a drug is administered orally, the primary site of absorption is typically the digestive tract [57]. The extent to which a substance is taken up by the human gastrointestinal tract is influenced by various parameters related to intestinal absorption. When the absorption rate of a molecule falls below 30%, it is considered to exhibit inadequate absorption efficacy (source: https://biosig.lab.uq.edu.au/pkcsm/theory) [58].

Compounds like Eserine (Standard) and Homoarecoline (CP2) exhibit high levels of intestinal absorption, indicating they might effectively pass through the intestines into the bloodstream. On the other hand, several compounds such as Dictyoquinazol C (CP21) and Mitoxantrone (CP24) show relatively lower levels of intestinal absorption, suggesting they might have limitations in their ability to pass through the intestinal barrier and get absorbed into the body’s circulation. Cytochrome P450 (CYP450) is a hepatic enzyme primarily responsible for the oxidation of xenobiotics, aiding in their elimination [59]. Numerous medications can be either activated or deactivated by various isoforms of cytochrome P450, such as CYP1A2, CYP2C19, CYP2C9, and CYP2D6. Therefore, maximizing the inhibitory potential of a chemical compound is crucial for effectively blocking the enzyme. Table presents the predicted outcomes for the compounds regarding their ability to inhibit a specific isoform of cytochrome P450. Cytochrome P450 enzymes play a crucial role in the processing of many drugs. However, inhibitors of P450 enzymes can significantly alter the way these compounds are metabolized, affecting their pharmacokinetics. Therefore, it is important to consider that the chemicals provided are likely substrates of cytochrome P450. Among the various isoforms, cytochrome P450 3A4 is primarily responsible for drug metabolism. The results in the table indicate whether the substances listed will undergo metabolism by cytochrome P450 or not. The clearance of a drug, which involves both hepatic clearance (metabolism in the liver and excretion via bile) and renal clearance (excretion via the kidneys), is evaluated using the proportionality constant CLtot [60].

Bioavailability is a crucial factor in determining appropriate dosage rates to achieve steady-state concentration [61]. S2 Table also presents the logarithmic values of expected total clearance (CLtot) of a drug measured in (ml/min/kg). The AMES toxicity test is commonly employed to assess a compound’s potential for inducing bacterial mutations. A positive result suggests that the material is mutagenic, which means it will function similarly to a carcinogenic agent. Majority of compounds in the library exhibit zero toxicity. These findings suggest that these compounds may possess a favorable safety profile for use in clinical trials. The findings align with those reported by Fang et al. (2014), who identified crucial interactions involving residues like Tyr124 and Phe338 in AChE inhibition [55].

#### 3.6.4. DFT and MESP studies.

**Fig 11:**
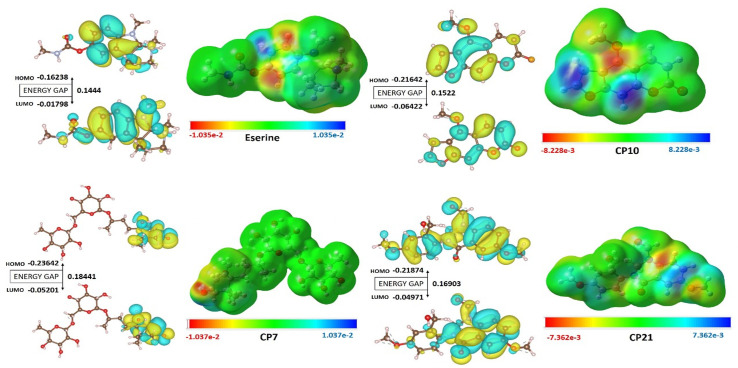
ESP structures (in solvent phases) formed by mapping of total density over electrostatic potential, and optimized structures of eserine, CP7, CP10, and CP21. Calculated HOMO and LUMO orbitals of potent derivatives at B3LYP/SVP level of DFT calculations for all selected ligands.

The Molecular Electrostatic Potential (MESP) mappings have conducted a comparative analysis of the electronic properties of acetylcholinesterase (ACHE) inhibitors, specifically targeting three prominent compounds: CP7, CP10, and CP21. As illustrated in Fig 11, these mappings delineate the unique electronic characteristics that facilitate specific biochemical interactions with ACHE. The studies were executed in aqueous settings to evaluate the CNS-related neuroprotective functions of these CP compounds under physiological conditions, providing a pertinent context for analyzing these prospective therapeutics.

The MESP mappings underscore areas of pronounced electronegative potential, identified by a deep red hue across all three compounds. These crucial zones indicate preferred sites for electrophilic attack, essential for robust molecular binding and ACHE inhibition. Detailed Mulliken population analysis reveals that the oxygen atom at position 7 in CP7 possesses an average Mulliken charge of −0.515147, marked as the most intensely red region on the MESP mapping. A similar pattern is observed in CP10, where the oxygen at position 13 has a Mulliken charge of −0.543046, augmenting its ability for hydrogen bonding at ACHE’s strategic hinge and solvent-exposed areas. CP21 also presents a negatively charged area at oxygen position 14 with a Mulliken charge of −0.576206, enhancing its hydrogen bonding potential with ACHE. Notably, CP7 exhibits more neutral regions (indicated in green), implying a potential for hydrophobic or van der Waals (vdW) interactions, while WTHA features a balanced mix of red and green areas, supporting various types of interactions. All these electrostatic chemical parameters are comparable with the standard Esserine, which displays two intensely electronegative sites in red at positions 5O and 8C, with Mulliken charges of −0.638766 and −0.291504 respectively.

This pivotal analysis is essential for determining the reactivity of the compounds. The HOMO-LUMO energy gap, indicative of a molecule’s kinetic stability, elucidates the energy differential between the highest occupied and lowest unoccupied molecular orbitals, enabling enhanced energy transfer within the molecule. The molecular surface plots of the HOMO and LUMO orbitals for eserine, CP7, CP10, and CP21 are showcased in Fig 11. The electron-acceptor potential of an inhibitor is mirrored by its LUMO value, while its HOMO value influences its electron-donating ability.

Table 7 succinctly offers a detailed summary of the computed quantum chemical descriptors for these compounds under aqueous conditions. DFT calculations have clarified crucial molecular characteristics of these ACHE inhibitors, unveiling their distinct electronic structures and reactivities.

**Table 7:** Parameters for DFT analysis of the most active compounds from the studied extract.

Parameters for DFT analysis											
Ligands	Dipole moment (Debye)	HOMO (a.u.)	LUMO (a.u.)	Energy Gap (ΔE_Gap_)	Ionization Potential (eV)	Electron affinity (eV)	Electronegativity χ (eV)	Electrochemical potential μ (eV)	Hardness η (eV)	Softness S (eV)	Electrophilicity ω (eV)
Eserine	19.5793	−0.16238	−0.01798	0.1444	4.42	0.49	2.45	−2.45	1.96	0.509	1.53
CP7	6.6844	−0.23642	−0.05201	0.18441	6.43	1.42	3.92	−3.92	2.51	0.398	3.07
CP10	9.551	−0.21642	−0.06422	0.1522	5.89	1.75	3.82	−3.82	2.07	0.483	3.52
CP21	4.7659	−0.21874	−0.04971	0.16903	5.95	1.35	3.65	−3.65	2.3	0.435	2.9

Eserine, in particular, displayed an exceptionally high dipole moment of 19.5793 Debye, presenting a unique electronic profile in contrast to CP7, CP10, and CP21, which exhibited considerably lower dipole moments of 6.6844, 9.551, and 4.7659 Debye, respectively. This differentiation suggests varied electron-donating capacities and interaction potentials with ACHE. Among the three chosen ligands, CP10 registered the highest dipole moment. The HOMO-LUMO energy gap analysis further showed that CP10 also mirrored the comparative disparity with standard eserine, indicating heightened reactivity and potential biological effectiveness. These insights not only enhance our comprehension of the molecular basis for inhibitory activity against ACHE but also underscore the imperative to fine-tune electronic and structural attributes for developing potent ACHE inhibitors. This comprehensive analysis lays the groundwork for future structure-activity relationship studies, which will inform the creation of novel inhibitors with enhanced therapeutic profiles.

Employing Koopman’s theorem, global reactivity parameters for ligands Eserine, CP7, CP10, and CP21 have been computed, demonstrating notable differences in their electronic properties as depicted in the data. Eserine is characterized by a moderate level of electronegativity (χ = 2.45 eV) and chemical potential (μ = −2.45 eV), paired with a relatively lower hardness (η = 1.96 eV). This level of hardness suggests a softer resistance to deformation of its electron cloud, which is also indicated by its higher global softness (σ = 0.509 eV^-1) and moderate electrophilicity index (ω = 1.53 eV). In contrast, CP7 exhibits a higher electronegativity (χ = 3.92 eV) and chemical potential (μ = −3.92 eV), coupled with the highest hardness (η = 2.51 eV) observed among the studied ligands. This pronounced hardness underscores a more robust resistance to electronic reconfiguration, paired with a lower global softness (σ = 0.398 eV^-1), which correlates with a higher capacity to attract additional electrons, as reflected in its electrophilicity index (ω = 3.07 eV). CP10 shows slightly lesser electronegativity (χ = 3.82 eV) and chemical potential (μ = −3.82 eV) than CP7, with a hardness (η = 2.07 eV) that suggests moderate resistance to electronic deformation. Its global softness (σ = 0.483 eV^-1) indicates a fair capacity for electron mobility, which is accompanied by a significant electrophilicity index (ω = 3.52 eV), pointing to its reactivity under electrophilic conditions.

CP21, with an electronegativity of 3.65 eV and a chemical potential of −3.65 eV, demonstrates a balance in its electronic properties. It features a hardness (η = 2.3 eV) that supports a moderate resistance to electronic shifts, paired with a softness (σ = 0.435 eV^-1) that enables some flexibility in electron distribution. CP21’s electrophilicity index (ω = 2.9 eV) signifies a balanced reactivity, potentially enhancing its utility in various chemical environments. These distinct parameters illuminate the underlying electronic behavior of these ligands, highlighting a spectrum of reactivity profiles that could influence their utility in different chemical contexts. A study by Mohammadi and Ghayeb (2018) provides a relevant comparison, emphasizing the importance of HOMO-LUMO gaps and global reactivity parameters in assessing the electronic properties of AChE inhibitors [62]. The study highlights similar findings regarding the significance of dipole moments, hardness, and softness in determining the reactivity and binding efficacy of AChE inhibitors.

#### 3.6.5. Dynamic simulations, comprehensive analysis of structural flexibility and stability.

In this study, molecular dynamics (MD) simulations and free energy calculations were utilized to investigate the binding modes and interaction mechanisms of the eserine inhibitor and CP21, targeting acetylcholinesterase (AChE) with differing efficacies. Notably, CP21 demonstrated an inhibitory capacity about twice that of eserine, as detailed in Fig 10A. A detailed comparison, shown in Fig 10B, highlights three significant structural changes in CP21 that substantially improve its AChE binding affinity relative to eserine. These changes include replacing the aliphatic chain on the benzene ring with a methyl-hydroxy benzene group, and substituting both pyrrolidine rings with a pyrimidine ring. Moreover, the interactions of methyl-hydroxyl groups connected to the benzene linked with the pyrimidine ring play a crucial role in enhancing AChE binding. Additionally, as shown in Figs 10A,10B, eserine, which shares structural similarities and comparable AChE inhibitory potency with CP21, provides a structural basis for this increased activity. Docking studies have shown that both inhibitors assume a similar curve-shaped conformation within the AChE ATP binding pocket. Consequently, CP21 and eserine were selected for further MD simulations and binding free energy studies to determine the critical structural features required for selective AChE inhibition, thus laying the groundwork for developing more effective AChE inhibitors.

**Fig 12:**
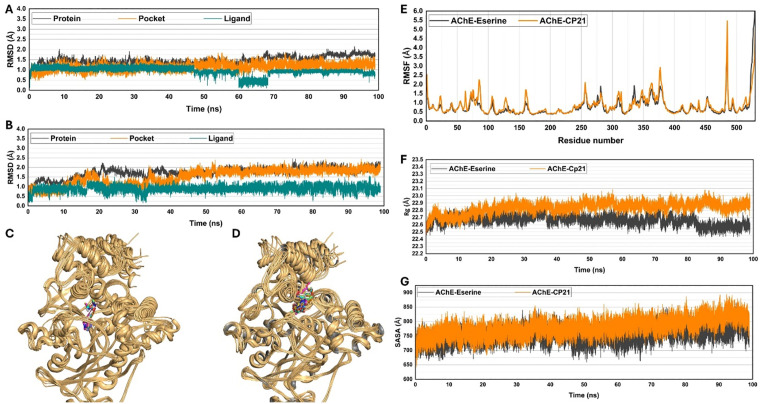
(A-B) Root-mean-square deviation (RMSD) curve for AChE-CP21 and AChE-Eserine structure (C) Ligand binding cavity in AChE with Eserine (D) Selected complex occupying the same binding site of AChE-CP21. **(E)** Root mean fluctuation (RMSF) curve for AChE-CP21 and their comparison to AChE-Eserine structure. **(F and G)** Plots for radius of gyration (ROG) and solvent accessible surface area (SASA) for both complexes.

To evaluate the dynamic stability of our systems and verify the effectiveness of the sampling method, we monitored the root-mean-square deviation (RMSD) from the initial structures over 100 ns of MD simulations. The RMSD evaluations indicated that all systems, including the AChE-CP21 and AChE-Eserine complexes, reached equilibrium within the first 5 ns. Notably, the RMSD values for the Cα atoms of the protein, the backbone atoms of the binding pocket, and the heavy atoms of the ligands stabilized at average values of about 1.5 Å, 1.3 Å, and 0.9 Å, respectively. These values are visually presented in Fig 12A and 12B, showcasing the stability of the systems, which forms a solid basis for further analyses such as hydrogen bonding free energy and energy decomposition based on conformations sampled from 5–100 ns. The consistency of these conformations was further validated by overlaying the coordinates of representative MD-simulated snapshots onto their initial structures, as depicted in Figs 12C and 12D. This structural assessment confirmed that all complexes, including AChE-CP21 and AChE-Eserine, maintained stability throughout the simulation, with all ligands retaining their initial conformation and essential hydrogen bonds with the CAS and PAS region residues intact. These results affirm the precision of our MD simulation outcomes for advancing binding free energy studies, providing valuable insights into the complex interaction mechanisms of these inhibitors with AChE.

The root-mean-square fluctuation (RMSF) analysis covering all ligand-protein interactions, as shown in Fig 12E, outlines the dynamic profiles and RMSF distribution across the protein structures of all evaluated systems. These dynamics exhibit consistent patterns, with three areas within the AChE structure CAS, PAS, and Mid Gorge (located between the CAS and PAS)—showing pronounced fluctuations. Specifically, the CAS, comprising residues 119–126, 480–490, and 447–458, displayed higher fluctuations in both non-bonded and bonded interactions with AChE, whereas the PAS region, encompassing residues 72–76 and 250–290, also exhibited significant but slightly lower fluctuations in systems bonded with AChE-CP21. This observation suggests that ligand binding increases the mobility of the Mid Gorge (residues 291–300), indicating enhanced flexibility between the CAS and PAS regions in the presence of AChE. Additionally, residues 480–490 in the CAS region exhibited the highest fluctuations in the AChE-Eserine and CP21 complexes. Despite the variability, the Mid Gorge and PAS regions generally followed a similar fluctuation pattern, whereas AChE-Eserine and CP21 complexes noted fluctuations in these loops. In contrast, the CAS, PAS, and Mid Gorge regions displayed stability across both AChE systems, emphasizing their crucial role in maintaining structural integrity upon ligand binding. Notably, the CAS residues in receptor-ligand bonded systems exhibited a heightened fluctuation amplitude compared to those in the non-bonded AChE configuration, highlighting the significant dynamic influence of ligand binding on the mobility of this critical region.

To assess the influence of ligand binding on the structural stability of the protein, the radius of gyration (Rg) of AChE was monitored during the simulation, as shown in Fig 12F. The average Rg values calculated for AChE bound to eserine and CP21 were approximately 22.5 Å and 22.8 Å, respectively, showing notable consistency. This stability suggests that the binding of these ligands does not cause significant alterations to the overall structure of the protein, such as unfolding or expansion. Moreover, the Rg values remained within a tight range of 22.4 Å to 22.9 Å throughout the simulation, reinforcing the strong structural integrity of the protein. These observations indicate that the interaction of eserine and CP21 with AChE maintains the enzyme’s native conformation, which is vital for their inhibitory function by preserving the structure of the active site needed for effective ligand recognition. Additional understanding was obtained from analyzing the solvent-accessible surface area (SASA), providing deeper insights into the dynamics between AChE and the investigated inhibitors, as illustrated in Fig 12G. The SASA of the complex with CP21 was roughly 750 Å², demonstrating a stable interaction within this complex. Remarkably, eserine displayed a stability similar to CP21, with average SASA values around 720 Å². The consistency in these measurements across both complexes highlights the effectiveness of these ligands as potential inhibitors of AChE. A study by Gharaghani et al. (2013) provides a relevant comparison, combining MD simulation and docking studies to explore the stability and binding interactions of AChE inhibitors [63]. This study also emphasizes the importance of hydrophobic interactions and structural stability in the enzyme-inhibitor complexes.

#### 3.6.6. Binding free energy analysis.

**Fig 13:**
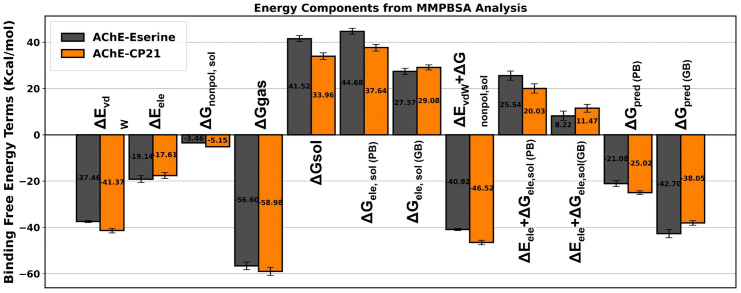
Binding free energy (Kcal/mol) terms of AChE-Eserine and AChE-CP21.

Given the system stability ascertained through RMSD fluctuations depicted in Fig 13, we extracted 10,000 snapshots extracted from last 10 ns of the stable MD simulation trajectories for binding free energy calculations. The binding affinities of the chosen compounds towards AChE were determined using both MM/PBSA and MM/GBSA methods. The predicted binding free energies (ΔG_pred (GB)_) for CP21 and Eserine using MM/GBSA were −38.05 and −41.12 kcal/mol, while MM/PBSA (ΔG_pred (PB)_) yielded −25.02 and −21.08 kcal/mol, respectively. These findings highlight the enhanced reliability of MM/GBSA and MM/PBSA methods over conventional docking, validating our computational pipeline for accurately estimating the binding efficiency of AChE inhibitors. The observed differences between ΔG_pred (GB)_ and ΔG_pred (PB)_ values arise from their distinct treatment of solvation models, where GB approximates the solvent reaction field, while PB solves the Poisson-Boltzmann equation more rigorously, often resulting in more conservative energy estimates. The MMGB/PBSA method’s ability to dissect the total binding free energy into its constituent components enhances our understanding of the ligand-receptor binding dynamics. As detailed in Fig 13, the polar solvation energies (ΔE_ele, sol_) exhibit positive values, offsetting the favorable electrostatic energies (ΔE_ele_) observed in the gas phase for both complexes. This results in the combined electrostatic contributions (ΔG_ele_ + ΔG_ele, sol_) being unfavorable for the formation of ligand-receptor complexes. Conversely, the *van der Waals (vdW)* interactions and nonpolar solvation energy (ΔE_*vdW*_ + ΔG_nonpol,sol_) contribute negative values, reinforcing the binding affinity of CP21 to AChE. Notably, the ΔE_*vdW*_ values surpass the ΔE_ele_ term for all examined systems, highlighting the critical role of optimizing *vdW* and nonpolar interactions in enhancing the inhibitory effectiveness of AChE inhibitors.

The presence of several hydrophobic residues, such as W286, Y124, F295, H447, Y341, V294, F338, and R296, aligns with the observation that hydrophobic interactions significantly influence binding efficiency. Despite the electrostatic contribution being less dominant compared to the *vdW* and nonpolar solvation contributions, it remains a pivotal factor in mediating interactions between AChE and inhibitors like CP21 and Eserine. Electrostatic interactions particularly enhance the binding effects with Eserine more than with CP21. Analysis of the AChE complexes further exemplifies the predominance of *vdW* interactions in modulating inhibitory potency, with the ΔE_*vdW*_ term showcasing significant negative values for AChE-CP21 (−41.37 kcal/mol) and AChE-Eserine (−37.46 kcal/mol) complexes. These findings emphasize that while electrostatic interactions contribute to the binding process, the *vdW* and hydrophobic interactions play a more substantial role in determining the inhibitory potential of AChE inhibitors. This analysis not only sheds light on the binding mechanisms but also offers strategic directions for designing more potent AChE inhibitors by focusing on enhancing van der Waals and hydrophobic interactions. A study by Hou et al. (2011) systematically evaluated the MM/PBSA and MM/GBSA methods for predicting binding free energies and provided insights into their accuracy and application in drug design. This study reported that MM/PBSA generally provides more accurate absolute binding free energies, while MM/GBSA is effective for ranking relative binding affinities [63].

CP21 was ranked not just based on docking scores, but also on convergence across numerous criteria. It replicated Eserine’s binding interactions, remained stable throughout 100 ns MD simulations (RMSD ~1.5 Å, Rg ~ 22.8 Å, SASA ~750 Å²), and had binding free energies comparable to the standard (MM/GBSA −38.05 vs. −41.12 kcal/mol; MM/PBSA −25.02 vs. −21.08 kcal/mol). Pharmacokinetic projections also backed its selection, since CP21 demonstrated adequate intestinal absorption, no CYP450 inhibition, low AMES toxicity, and simplicity of synthesis. The projected BBB permeability (−0.667) was lower than Eserine’s (0.154), but still within the moderate range (−1 < log BB < 0.3) and higher than CP1 (−1.026). Overall, CP21 was superior to the other tested compounds and, although not exceeding Eserine, demonstrated substantially similar performance, validating its selection as the most promising option.

#### 3.6.7. Limitations.

This study has sme limitations. Firstly, it relies on a haloperidol-induced model of PD, which, though effective for inducing motor deficits and catalepsy, does not fully replicate the progressive development of neurodegeneration of dopaminergic neurons, which is characteristic of idiopathic PD. Secondly, although ADMET predictions for key compounds like CP21 are promising, its relatively lower intestinal absorption highlights a potential challenge for oral bioavailability that must be addressed in future formulations. Moreover, these preclinical findings require validation in clinical trials to establish efficacy and safety in humans.

## 4. Conclusion

The phytochemical analysis of AJME revealed significant antioxidant properties, with 63.378% DPPH inhibition at 1 mg/ml and 60.78% acetylcholinesterase (AChE) inhibition (IC_50_ of 200.81 μg/mL*). In vivo* studies on haloperidol-induced Parkinson’s disease in rats demonstrated AJME’s anti-Parkinsonian potential. Behavioral tests showed significant improvements in locomotor activity, exploratory behaviors, and memory. AJME also enhanced oxidative stress parameters (SOD, CAT, GSH) and reduced malondialdehyde (MDA) and nitrite levels, while neurotransmitter levels (dopamine, noradrenaline, serotonin) significantly recovered, mitigating neuronal degeneration. *In silico* studies identified CP21 as a potent ligand with a docking score of −9.091 kcal/mol. Molecular dynamics simulations and binding free energy calculations confirmed the stability and strong inhibitory potential of AJME compounds. AJME shows promise as a therapeutic candidate for Parkinson’s disease, improving oxidative stress parameters, neurotransmitter levels, and neuronal integrity. While AJME shows promising preclinical results, it is important to note that these findings are based solely on animal models, and clinical applicability has not yet been demonstrated. Further research and clinical trials are needed to explore its full potential in Parkinson’s disease management.

## Supporting information

S1 Fig Count of residue interaction with Ligands. (TIF)

S1 Table Compounds identified from Liquid Chromatography Mass Spectrometry (LCMS) Analysis. (DOCX)

S2 Table Pharmacokinetic Properties of Compounds Identified from LCMS. (DOCX)

S3 Table Preliminary phytochemical screening tests used to identify secondary metabolites in the extract. (DOCX)

S4 Table Experimental groups and the respective treatments used in the Parkinsonian model study. (DOCX)
