# Correction: Unveiling the biochemical potential of *Acacia jacquemontii* as a therapeutic agent in parkinson’s disease: A multi-model *in Vitro, In Vivo, and In Silico* Study

**DOI:** 10.1371/journal.pone.0346807

**Published:** 2026-04-08

**Authors:** Andleeb Asghar, Tahir Ali Chohan, Aisha Qayyum, Sibghat Mansoor Rana, Khuram Ashfaq, Hafiz Muhammad Mazhar Asjad, Talha Ali Chohan, Abdulwahab Alamri, Ahmed Alsolami, Fawwaz F. Alshammrie, Hammad Saleem, Sirajudheen Anwar

The following information is missing from the Funding statement: The authors acknowledge the Scientific Research Deanship at the University of Hail, Saudi Arabia, through project number RG-23149.
