# Supplementary figures and images for: Unveiling the biochemical potential of Acacia jacquemontii as a therapeutic agent in parkinson’s disease: A multi-model in Vitro, In Vivo, and In Silico Study
Source: PLoS One. 2026 Feb 19;21(2):e0334312. doi: 10.1371/journal.pone.0334312 (PMC12919844; doi:10.1371/journal.pone.0334312)

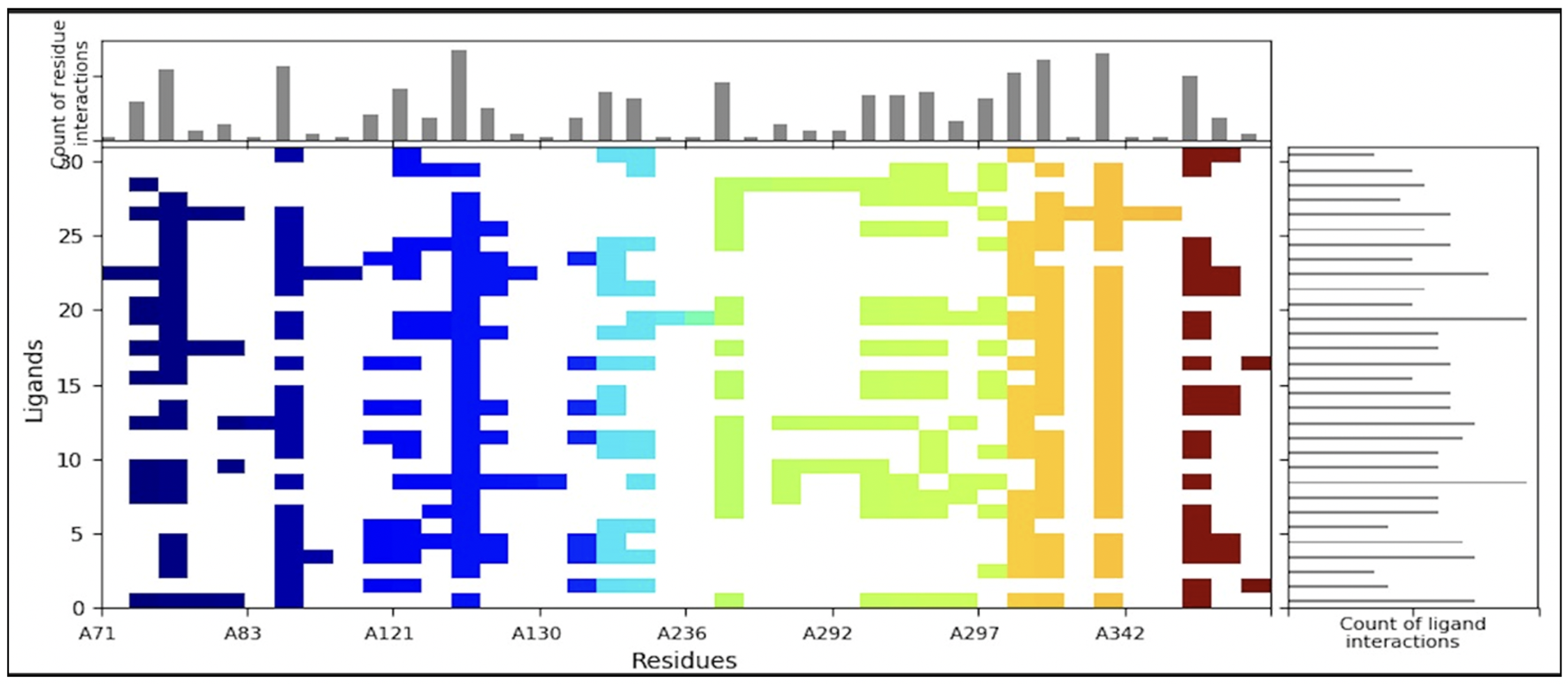

Supplement: S1 Fig — (TIF) [file pone.0334312.s001.tif]
