# Supplementary material for: Unveiling the biochemical potential of Acacia jacquemontii as a therapeutic agent in parkinson’s disease: A multi-model in Vitro, In Vivo, and In Silico Study
Source: PLoS One. 2026 Feb 19;21(2):e0334312. doi: 10.1371/journal.pone.0334312 (PMC12919844; doi:10.1371/journal.pone.0334312)
Supplement: S1 Table — (DOCX) [file pone.0334312.s002.docx]

**Table SP1: Compounds identified from** **Liquid Chromatography Mass Spectrometry (LCMS) Analysis**

| **Compound Label** | **RT** | **Mass** | **DB Formula** | **Chemical class** | **DB Diff (ppm)** | **Hits  (DB)** |
| --- | --- | --- | --- | --- | --- | --- |
| **N-Methylcalystegine B2 (1)** | **0.699** | 189.1008 | C_8_ H_15_ N O_4_ | Pyrrolizidine | -3.92 | 10 |
| **Homoarecoline (2)** | 0.784 | 169.1105 | C_9_ H_15_ N O_2_ | Alkaloid | -1.45 | 10 |
| **D-Proline (3)** | 1.475 | 115.0638 | C_5_ H_9_ N O_2_ | Amino Acid | -3.86 | 6 |
| **PF-750 (4)** | 4.8 | 345.1844 | C_22_ H_23_ N_3_ O | Carboxamide | -0.8 | 1 |
| **Amitraz (5)** | 5.862 | 293.1905 | C_19_ H_23_ N_3_ | Formamidine | -4.44 | 1 |
| **Indoramin (6)** | 7.672 | 347.2 | C_22_ H_25_ N_3_ O | Tryptamine | -0.65 | 1 |
| **Scandenin (7)** | 9.794 | 434.1749 | C_26_ H_26_ O_6_ | Coumarin | -4.56 | 7 |
| **Loperamide (8)** | 10.48 | 476.223 | C_29_ H_33_ Cl N_2_ O_2_ | Alkaloid | 0.06 | 1 |
| **7,8-Dihydrovomifoliol 9- [rhamnosyl-(1->6)-glucoside] (9)** | 11.109 | 534.2677 | C_25_ H_42_ O_12_ | Glycoside | -0.11 | 2 |
| **Bergapten (10)** | 0.643 | 216.042 | C_12_ H_8_ O_4_ | Psoralen | 1.31 | 9 |
| **3,3-Dimethyl-1,2-dithiolane (11)** | 0.746 | 134.0234 | C_5_ H_10_ S_2_ | Organosulfur | -7.66 | 3 |
| **Oleandrose (12)** | 4.794 | 162.0897 | C_7_ H_14_ O_4_ | Carbohydrate | -2.91 | 4 |
| **1-O-p-Coumaroyl-(b D-glucose 6-O-sulfate) (13)** | 7.635 | 406.0588 | C_15_ H_18_ O_11_ S | Glycoside | -4.5 | 1 |
| **Benfuracarb (14)** | 7.689 | 410.1889 | C_20_ H_30_ N_2_ O_5_ S | Coumaran | -3.39 | 4 |
| **Dihydroisolysergic acid II (15)** | 8.223 | 270.1375 | C_16_ H_18_ N_2_ O_2_ | Ergoline Alkaloid | -2.46 | 1 |
| **14,19- Dihydroaspidospermatine (16)** | 8.666 | 340.2145 | C_21_ H_28_ N_2_ O_2_ | Alkaloid | 1.62 | 10 |
| **Lupanyl Acid (17)** | 9.403 | 252.1827 | C_14_ H_24_ N_2_ O_2_ | Quinic Acid | 4.08 | 1 |
| **Leoidin Dimethyl Ether (18)** | 9.548 | 440.0448 | C_20_ H_18_ Cl_2_ O_7_ | Ether | -4.07 | 1 |
| **4-(3-Methylbut-2- enyl)-L-tryptophan (19)** | 9.611 | 272.1536 | C_16_ H_20_ N_2_ O_2_ | Tryptophan | -4.16 | 4 |
| **Carbinoxamine (20)** | 9.657 | 290.1196 | C_16_ H_19_ Cl N_2_ O | Alkylamine | -3.57 | 4 |
| **Dictyoquinazol C (21)** | 9.662 | 342.1224 | C_18_ H_18_ N_2_ O_5_ | Alkaloid | -2.32 | 10 |
| **(R*,S*)-4-[1-Ethyl-2-(4-fluorophenyl)butyl]phenol (22)** | 9.724 | 272.1571 | C_18_ H_21_ F O | Phenol | 2.03 | 3 |
| **Clozapine-N-Oxide (23)** | 9.791 | 342.1253 | C_18_ H_19_ Cl N_4_ O | Dibenzodiazepine | -1.74 | 1 |
| **Mitoxantrone (24)** | 9.791 | 444.2032 | C_22_ H_28_ N_4_ O_6_ | Anthracenediones | -5.3 | 1 |
| **3-Quinolinecarboxylic acid, 7- amino-1-ethyl-6-fluoro-1,4- dihydro-4-oxo (25)** | 9.793 | 250.0759 | C_12_ H_11_ F N_2_ O_3_ | Quinoline | -2.25 | 10 |
| **Dihydro-4-oxo- Cpd 57: Idarubicin (26)** | 9.794 | 497.1696 | C_26_ H_27_ N O_9_ | Quinoline | -2.11 | 7 |
| **Ferulic acid (27)** | 10.011 | 194.0586 | C_10_ H_10_ O_4_ | Cinnamic Acids | -3.64 | 10 |
| **Penicilloic acid V (28)** | 10.342 | 368.1056 | C_16_ H_20_ N_2_ O_6_ S | Beta-Lactams | -3.78 | 3 |
| **Antimycin A (29)** | 11.302 | 534.2588 | C_27_ H_38_ N_2_ O_9_ | Depsipeptides | -1.98 | 1 |
| **Glycylalanylprolylmethiony phenylalanylvalinamide (30)** | 11.857 | 619.3183 | C_29_ H_45_ N_7_ O_6_ S | Peptide | -5.04 | 1 |
