# Supplementary material for: Unveiling the biochemical potential of Acacia jacquemontii as a therapeutic agent in parkinson’s disease: A multi-model in Vitro, In Vivo, and In Silico Study
Source: PLoS One. 2026 Feb 19;21(2):e0334312. doi: 10.1371/journal.pone.0334312 (PMC12919844; doi:10.1371/journal.pone.0334312)
Supplement: S2 Table — (DOCX) [file pone.0334312.s003.docx]

**Table SP2: Pharmacokinetic Properties of Compounds Identified from LCMS**

| **Sr. No.** | **Compound label** | **Docking scores** | **Pharmacokinetics** | | | | | | | | | | **Synthetic accessibility** | |
| --- | --- | --- | --- | --- | --- | --- | --- | --- | --- | --- | --- | --- | --- | --- |
|  |  |  | **Absorption** | **Metabolism** | | | | | | | **Excretion** | **Toxicity** |  |  |
|  |  |  | **Intestinal absorption**  **(Human)** | **CYP** | | | | | | | **Total Clearance** | **AMES**  **Toxicity** |  |  |
|  |  |  | Numeric (% absorbed) | 2D6  Sub | 3A4  sub | 1A2  inh | 2C19  Inh | 2C9  inh | 2D6  inh | 3A4  inh | Numeric (log ml/min/kg) | Categorical  (yes/no) | Numeric |  |
| **1** | Standard | -9.512 | 91.576 | No | Yes | No | No | No | Yes | No | 0.563 | No | 3.38 |  |
| **2** | CP1 | -10.016 | 55.434 | No | No | No | No | No | No | No | 0.786 | No | 4.13 |  |
| **3** | CP2 | -5.938 | 95.916 | No | No | No | No | No | No | No | 0.824 | Yes | 2.78 |  |
| **4** | CP3 | -5.607 | 87.288 | No | No | No | No | No | No | No | 0.6 | No | 1.54 |  |
| **5** | CP4 | -7.726 | 90.83 | Yes | Yes | Yes | Yes | Yes | No | Yes | 0.399 | No | 2.49 |  |
| **6** | CP5 | -8.279 | 91.244 | No | Yes | Yes | Yes | Yes | Yes | No | 0.533 | No | 3.07 |  |
| **7** | CP6 | -6.327 | 92.333 | Yes | Yes | Yes | No | No | Yes | Yes | 1.186 | No | 2.36 |  |
| **8** | CP7 | -8.433 | 16.953 | No | No | No | No | No | No | No | 1.263 | No | 6.81 |  |
| **9** | CP8 | -5.838 | 92.729 | Yes | Yes | No | No | No | Yes | No | 0.768 | Yes | 3.17 |  |
| **10** | CP9 | -7.698 | 96.057 | No | Yes | No | Yes | Yes | No | Yes | 0.607 | No | 4.44 |  |
| **11** | CP10 | -6.406 | 98.344 | No | No | Yes | Yes | Yes | Yes | Yes | 0.805 | Yes | 2.90 |  |
| **12** | CP11 | -5.08 | 94.255 | No | No | No | No | No | No | No | 0.193 | No | 2.55 |  |
| **13** | CP12 | -4.262 | 72.547 | No | No | No | No | No | No | No | 0.92 | No | 2.95 |  |
| **14** | CP13 | -8.826 | 16.458 | No | No | No | No | No | No | No | 0.319 | No | 4.77 |  |
| **15** | CP14 | -7.235 | 94.319 | No | Yes | No | Yes | No | No | Yes | 0.336 | No | 3.77 |  |
| **16** | CP15 | -7.884 | 93.336 | No | Yes | No | No | No | Yes | No | 0.902 | No | 4.11 |  |
| **17** | CP16 | -7.788 | 97.376 | Yes | Yes | No | No | No | No | No | 0.64 | No | 5.13 |  |
| **18** | CP17 | -8.285 | 91.051 | No | Yes | No | No | No | No | No | 1.071 | No | 4.02 |  |
| **19** | CP18 | -7.444 | 97.218 | No | Yes | No | No | Yes | No | Yes | 0.447 | No | 3.72 |  |
| **20** | CP19 | -7.312 | 91.52 | Yes | No | No | No | No | No | No | 0.658 | No | 2.89 |  |
| **21** | CP20 | -7.081 | 95.291 | No | Yes | No | No | No | Yes | No | 0.797 | No | 2.98 |  |
| **22** | CP21 | -9.091 | 98.471 | No | Yes | No | Yes | No | No | No | 0.281 | No | 2.81 |  |
| **23** | CP22 | -7.58 | 94.397 | No | No | No | No | Yes | Yes | Yes | 0.117 | No | 2.64 |  |
| **24** | CP23 | -6.863 | 93.237 | No | Yes | Yes | Yes | No | No | No | 0.865 | No | 4.20 |  |
| **25** | CP24 | -7.985 | 32.338 | No | No | Yes | No | No | No | No | 1.423 | Yes | 3.61 |  |
| **26** | CP25 | -7.168 | 97.12 | No | No | No | No | No | No | No | 0.567 | No | 1.96 |  |
| **27** | CP26 | -8.65 | 62.418 | No | No | Yes | No | No | No | No | 0.978 | Yes | 5.63 |  |
| **28** | CP27 | -5.309 | 95.598 | No | No | No | No | No | No | No | 0.641 | No | 1.93 |  |
| **29** | CP28 | -6.331 | 26.948 | Yes | No | No | No | No | No | No | 0.193 | No | 4.08 |  |
| **30** | CP29 | -6.316 | 64.305 | No | Yes | No | No | No | No | Yes | 0.856 | No | 5.76 |  |
| **31** | CP30 | -8.834 | 26.291 | No | No | No | No | No | No | No | 0.55 | No | 4.08 |  |
