# Supplementary material for: Unveiling the biochemical potential of Acacia jacquemontii as a therapeutic agent in parkinson’s disease: A multi-model in Vitro, In Vivo, and In Silico Study
Source: PLoS One. 2026 Feb 19;21(2):e0334312. doi: 10.1371/journal.pone.0334312 (PMC12919844; doi:10.1371/journal.pone.0334312)
Supplement: S3 Table — (DOCX) [file pone.0334312.s004.docx]

**Table SP3: Preliminary phytochemical screening tests used to identify secondary metabolites in the extract.**

| **Secondary Metabolite** | **Test Used** |
| --- | --- |
| **Anthraquinones** | Borntrager's test |
| **Tannins** | FeCl₃ test |
| **Alkaloids** | Dragendorff's test |
| **Cardioactive Glycosides** | Keller-Kiliani test |
| **Saponins** | Froth test |
| **Flavonoids** | Alkaline reagent test |
