# Supplementary material for: Unveiling the biochemical potential of Acacia jacquemontii as a therapeutic agent in parkinson’s disease: A multi-model in Vitro, In Vivo, and In Silico Study
Source: PLoS One. 2026 Feb 19;21(2):e0334312. doi: 10.1371/journal.pone.0334312 (PMC12919844; doi:10.1371/journal.pone.0334312)
Supplement: S4 Table — (DOCX) [file pone.0334312.s005.docx]

**Table SP4: Experimental groups and the respective treatments used in the Parkinsonian model study.**

| **Group** | **Treatment** |
| --- | --- |
| **Group 1 (Control)** | Vehicle (Saline) |
| **Group 2 (Disease Control)** | Haloperidol (1 mg/kg, intraperitoneal injection) |
| **Group 3 (Conventional Treatment)** | Levodopa (100 mg/kg) + Carbidopa (25 mg/kg) |
| **Group 4 (AJME Treatment)** | AJME (200 mg/kg) |
| **Group 5 (AJME Treatment)** | AJME (400 mg/kg) |
| **Group 6 (AJME Treatment)** | AJME (600 mg/kg) |
